# The effect of culture on the benefits of awake prone positioning for adults with COVID-19 acute respiratory distress syndrome: A systematic review and meta-analysis

**DOI:** 10.1097/EA9.0000000000000068

**Published:** 2025-02-05

**Authors:** Sowmyashree Kota Karanth, Saajid Z. Azhar, Maria J. Corrales-Martinez, Vijay Krishnamoorthy, Pattrapun T. Wongsripuemtet, Julien Cobert, Mona Hashemaghaie, Karthik Raghunathan

**Affiliations:** From the Department of Anesthesiology, Duke University, Durham, North Carolina (SKK, MJC-M, VK, PTW, MH, KR), Department of Emergency Medicine, Louisiana State University Health Sciences Center Shreveport, Shreveport, Lousiana (SZA) and Department of Anesthesiology, University of California, San Francisco, California, USA (JC)

## Abstract

**BACKGROUND:**

Randomised controlled trials (RCTs) conducted early during the pandemic showed that awake prone positioning (APP) significantly reduced the risk of intubation among adults with COVID-19 acute respiratory distress syndrome (ARDS), but more recent studies have questioned this benefit. We hypothesise that the effects of APP may vary with the national Power Distance Index (PDI), a measure of hierarchy in local culture.

**OBJECTIVE:**

To conduct a meta-analysis examining the effects of APP in adults with COVID-19 ARDS and examine whether effects differ between nations with a PDI less than 80 versus at least 80 (low versus high deference to authority).

**DESIGN:**

Systematic review and meta-analysis of RCTs.

**DATA SOURCES:**

Cumulated Index to Nursing and Allied Health Literature (CINAHL), the Cochrane Library, Embase, Medline and Scopus were searched to November 2024.

**ELIGIBILITY CRITERIA:**

All RCTs that compared APP with standard care in adults with COVID-19-related ARDS or Acute Hypoxaemic Respiratory Failure (AHRF) were included.

**RESULTS:**

Twenty-two RCTs were identified with 3615 patients having valid data. APP reduced the risk of intubation [relative risk (RR) 0.80, 95% confidence interval (CI), 0.72 to 0.90]. Effects were greater in nations with a PDI at least 80 (RR 0.67, 95% CI, 0.54 to 0.82), and there was equipoise in nations with a PDI less than 80 (RR 0.89, 95% CI, 0.75 to 1.05). Intubation rates in the high PDI nations decreased from 32.3% (*n* = 512) with standard care to 21.2% (*n* = 508) with APP. The reduction in intubations with APP was less pronounced in nations with low PDI, from 20.1% (*n* = 1012) with standard care to 17.1% (*n* = 1084). The risk of mortality reduced with APP (RR 0.86, 95% CI, 0.74 to 0.99). Fidelity of APP, specifically, adherence to the recommended duration, was higher in nations with PDI at least 80 (*P* = 0.04).

**CONCLUSION:**

APP reduces the risk of intubation and mortality, but the significance of this benefit varies with the cultural context. Effects are strong in nations with a higher PDI, where intubation rates are lower and adherence to APP higher.

## Graphical abstract

**Figure:**
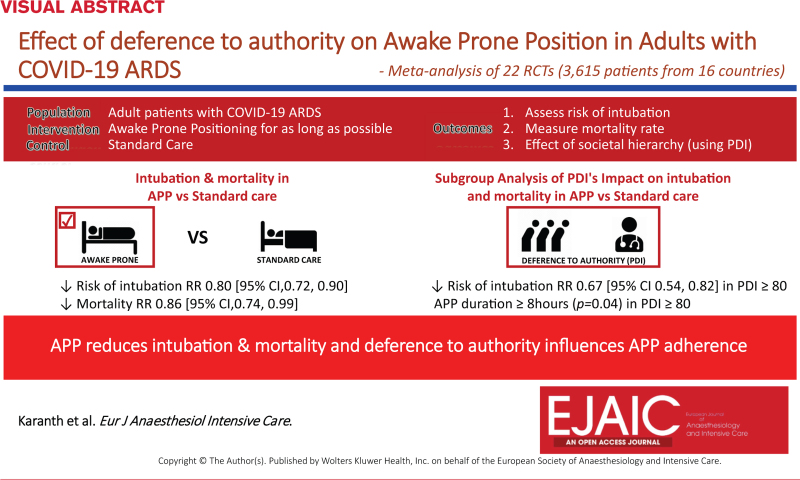

**KEY POINTS**

- Recent RCTs question the potential benefits of awake prone positioning in reducing intubation rates among adults with COVID-19 acute respiratory distress syndrome.
- In this analysis of 22 RCTs, awake prone positioning reduced rates of intubation (RR 0.80, 95% CI, 0.72 to 0.90) and mortality (RR 0.86, 95% CI, 0.74 to 0.99). The reduction in intubation was significantly greater in countries with a higher Power Distance Index (PDI ≥ 80) (RR 0.67, 95% CI, 0.54 to 0.82), and probably reflects greater hierarchy in physician–patient interactions and increased severity of illness.
- Awake prone positioning is a safe intervention with no major complications, and barriers to treatment fidelity must be determined.

## Introduction

Guidelines strongly recommend the prone position for the treatment of patients who are mechanically ventilated because of acute hypoxaemic respiratory failure (AHRF) with moderate to severe acute respiratory distress syndrome (ARDS).^1,2^ Awake prone positioning (APP) is a self-initiated intervention for nonintubated patients with ARDS.^3–6^ The intent is to improve oxygenation and avoid intubation by increasing alveolar recruitment in the larger posterior dependent areas of the lung, increasing functional residual capacity and decreasing lung injury.^3^ Earlier meta-analyses during the coronavirus disease 2019 (COVID-19) pandemic highlighted the advantages of APP.^4–6^ However, more recent studies have yielded mixed results. RCTs in various countries have differed in the duration of APP, reporting reduced intubation rates^7^ to no significant benefits,^8^ and even termination due to futility.^9^ A nonrandomised study in the United States even reported an increase in adverse outcomes.^10^

Why do some countries have more successful outcomes with APP? A meta-analysis published in early 2024 noted that benefits from APP are observed when the position was maintained for more than 8 h per day in the intensive care unit (ICU).^11^ We propose that the differences in the adoption and effectiveness of APP are probably influenced by variability in human behaviour, driven by national culture, which can be measured by Power Distance Indices (PDI).^12^

The PDI, a concept coined by the Dutch Psychologist Geert Hofstede, refers to the extent to which less powerful individuals ‘accept and expect that power is distributed unequally’.^13^ The PDI score ranges from 1 to 100, with scores at least 80 considered as the threshold for significant societal hierarchy.^14^ Studies have found a negative association between the PDI and morbidity and mortality due to COVID-19.^15,16^ The spread of COVID-19 in the early stages of the pandemic was slower in countries with high PDIs because of respect for authority and greater compliance with rules.^16^ Consequently, it is proposed that this deference to leaders in countries with high PDIs may translate into greater APP adherence and improved outcomes, as patients are more likely to follow instructions from physicians, nurses or family members. In contrast, patients in countries with lower PDIs, like the United States and Canada, may be less inclined to adopt new treatments, viewing them as optional rather than obligatory. This reluctance could hinder APP adoption and its associated benefits. Therefore, we hypothesise that APP reduces intubation rates and mortality, and the benefits of APP are greater in countries with higher PDIs, and that the duration of APP is longer in them (PDI <80 versus ≥80). We conducted a new meta-analysis, as an earlier one included an RCT twice, influencing the robustness of their conclusions,^17^ and four new RCTs^18–21^ were available. We also sought to stratify RCTs by the PDIs of the countries where they were conducted.

## Methods

This systematic review and meta-analysis was conducted in accordance with the Preferred Reporting Items for Systematic Reviews and Meta-analyses (PRISMA) guidelines.^22^ The protocol was registered with the International Prospective Register of Systematic Reviews (PROSPERO, registration no. CRD42023428236) and OSF registries (https://doi.org/10.17605/OSF.IO/3JKZ8).

### Search strategy and study selection

The Cochrane Library [Cochrane Central Register of Controlled Trials (CENTRAL) interface], Cumulated Index to Nursing and Allied Health Literature (CINAHL), EMBASE including MEDLINE (PubMed interface) and Scopus databases were searched from inception to 22 November 2024. A search strategy that included MeSH terms and keywords related to ARDS or AHRF and Self-Prone, Proning, or Awake Prone Position modified according to the database was used (Supplement – search strategy). English language RCTs comparing awake prone position with standard care (no APP) in patients at least 18 years of age with ARDS or AHRF were included. Non-RCTs, grey literature, other meta-analyses, observational studies, studies involving neonates and children and studies involving prone positioning of intubated patients were excluded. Two reviewers independently screened titles and abstracts of studies identified by database searches. After applying the inclusion and exclusion criteria, selected studies underwent full-text review.

### Outcomes

The primary outcomes were intubation and mortality up to the longest follow-up, most often 28 or 30 days, in patients with and without APP. The secondary outcomes were the association between PDI less than 80 and at least 80 countries and APP duration (<8 h versus ≥8 h), escalation of respiratory support (change from baseline oxygen delivery modality to higher modality such as high-flow oxygen, continuous positive airway pressure, noninvasive ventilation, hospital length of stay, need for ICU admission, changes in oxygenation (SpO_2_:FiO_2_) and adverse events as defined by the included trials.

### Data extraction

Two authors independently extracted the following data from the included trials: meta-data including first author, publication year, the country where the RCTs were conducted, study setting, the number of participants, inclusion criteria, characteristics of patients included in the trials (age, sex, ethnicity, BMI and comorbidities), intervention characteristics (the oxygen delivery modality, SpO_2_/FiO_2_, mean APP duration, target APP), control group standard details, outcomes and conclusions. For categorical data, outcomes were extracted as the ratio of the number of participants who experienced the outcome to the total number assessed. For continuous data, mean with standard deviation or medians with interquartile range were extracted.

### Bias risk assessment

The risk of bias for all included trials was assessed using the Cochrane Collaboration Risk of Bias tool.^23^ Two reviewers independently assessed and classified trials as high or low risk, or unclear, in the following domains: the randomisation process, allocation concealment, participant and personnel blinding, outcome assessment blinding, incomplete outcome data, selective reporting and other biases.

Discrepancies in the study selection, data extraction and bias risk assessment were resolved by discussion with a third reviewer.

### Statistical analysis

**Fig. 1:**
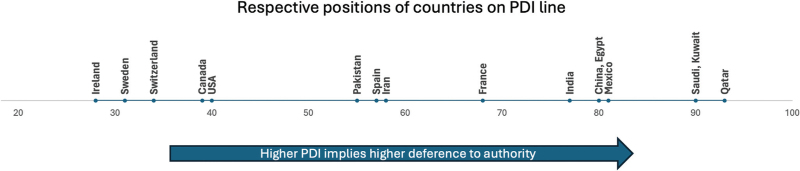
Respective positions of countries on Power Distance Index line.

Effect heterogeneity was examined by stratification of the analysis of primary outcomes according to national PDIs (≥80 versus <80). The PDIs, which is the degree to which individuals with less authority within a country's organisations accept uneven power distribution, for the countries involved were obtained from The Culture Factor Group (Fig. 1) (https://www.hofstede-insights.com/country-comparison-tool; accessed June 2024).^24^ Random effect models were used. The outcomes were reported as a relative risk (RR) ratio with a 95% CI for effect estimates (categorical data) and a mean difference with a 95% CI for continuous variables. The results were presented as Forest plots. Whenever possible, intention-to-treat data was considered. The percentage of total variance due to trial heterogeneity was assessed by visually inspecting the Forest plot and using the *I*^2^ statistic. *I*^ 2^ less than 60% was considered to represent low heterogeneity, and *I*^2^ at least 60% moderate or high heterogeneity. *P* values less than 0.05 were considered statistically significant for Forest plots and *P* values less than 0.1 were considered significant for tests of subgroup differences.^24^ Funnel plots were used to assess publication bias. To ensure that the conclusions were representative and robust, sensitivity analyses were performed by excluding trials with unclear and high risks of bias. We also performed a subgroup analysis for the outcome intubation according to the median duration of APP observed. We classified the groups as at least 8 h per day versus less than 8 h per day, as used by a previous meta-analysis.^11^ Certainty of evidence was assessed and reported using the Grading of Recommendations, Assessment, Development and Evaluation (GRADE) approach for all outcomes.^25^ The meta-analysis was performed using the Review Manager 5.4 software, abiding by the recommendations given in the *Cochrane Handbook of Systematic Reviews and Interventions*.^23^

## Results

**Fig. 2:**
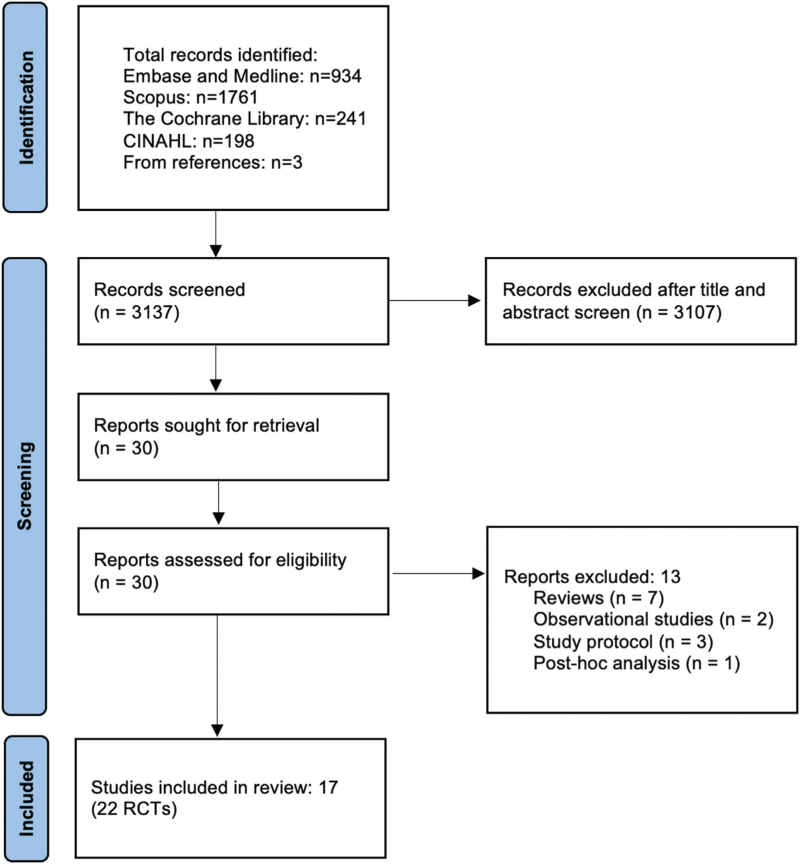
Preferred Reporting Items for Systematic Reviews and Meta-analyses flow diagram.

After the screening process,^7–9,18–21,26–35^ 17 studies including a total of 22 RCTs with 3615 patients were included in this meta-analysis (Fig. 2).

### Trial characteristics

The data extracted from trials, median APP duration, conclusions and PDIs are shown in Table 1 and Supplemental Data Tables 1 and 2. Of the 22 trials, six were of an international meta-trial, which reported results separately and in aggregate.^7^ Eight were multicentre studies.^7–9,20,21,30,33,34^ Two trials were cluster-randomised trials, whereas the rest were traditional RCTs.^32,35^ Six trials were terminated early, four because of a declining number of cases^20,32–34^ and two because of protocol nonadherence.^9,31^ Women comprised 31.1% of the intervention group and 34.3% of the control group. Ten trials were set in the ward,^9,18,20,26,27,29,31–33,35^ five in ICU^7,19,28,30^ and seven had mixed setting (ward and ICU).^7,8,21,32^ The target duration varied from four sessions of 1 to 2 h per day^9,29^ to 16 h per day^34^ or as long as tolerated.^7,31^ Two trials allowed patients to adopt both lateral position and prone position.^29,31^ Outcome data reporting was complete for all included RCTs, with no evidence of selective reporting of results. All RCTs except two^29,32^ described the randomisation process. Due to the nature of the intervention, APP, patients and observers were not blinded.

**Table 1:** Characteristics of the included randomised control trials and power distance indices

First author, year	Country	Power Distance Index	Setting	Number of participants in the intervention group	Number of participants in the control group	Enrollment location	Target Awake prone positioning duration	Primary outcome(s)	Median awake prone positioning duration [IQR], mean ± SD (intervention)	Median awake prone positioning duration [IQR] (control)
Harris,^20^ 2024	Qatar	93	Multi-centre	31	30	Not applicable	Up to 3 h per session with three sessions per day	Escalation of oxygen support from simple supplementary oxygen to non-invasive ventilation, high flow nasal cannula, or invasive mechanical ventilation; or from non-invasive mechanical ventilation or high flow nasal cannula to invasive mechanical ventilation by 30 days	18 h	0.3 h
Alhazzani,^8^ 2022	Canada, Kuwait, Saudi Arabia and the United States	39 (Canada), 90 (Kuwait), 72 (Saudi Arabia), 40 (United States)	Multicentre	205	195	High-density unit and intensive care unit	8 to 10 h per day with two to three breaks (one to two hours per break) as needed	Endotracheal Intubation within 30 days of randomisation	4.8 [1.8 to 8] h per day for up to 4 days	0 [0] h per day for up to 4 days
Ehrmann,^7^ 2021	Mexico	81	Multicentre	216	214	Ward, high dependency unit, and intensive care unit	At least 16 h per day, if possible	Endotracheal intubation or death within 4 weeks of randomisation	8.6 [6.1 to 11.4] h per day for up to 2 weeks	0 h per day
Liu,^21^ 2024	China	80	Multicentre	205	205	Ward, ICU/intermediate care unit	12 h per day	Endotracheal intubation within 4 weeks	12 [12 to 14] h per day (up to 1 week after randomisation)	5 [2 to 8] h per day
Nasrallah,^19^ 2023	Egypt	80	Multicentre	45	45	Intensive care unit	At least 30 min with high flow nasal cannula, and if tolerated well, a total of 8 h per day	Improvement or worsening of conditioning proceeding to intubation	Not applicable	Not applicable
Gad,^29^ 2021	Egypt	80	Single centre	15	15	Ward	1 to 2 h sessions 3 h apart during waking hours	1. Improvement in oxygenation (arterial oxygen saturation greater than 95% and arterial partial pressure of oxygen to fraction of inspired oxygen ratio greater than 200 mmHg) 2. Intubation within three days	Not applicable	Not applicable
Gopalakrishnan,^18^ 2023	India	77	Single-centre	257	245	High dependency unit	6 to 8 h per day	1. In-hospital mortality 2. Mortality, including follow-up period 3. Need for mechanical ventilation	4.3 ± 2.96	Not measured
Jayakumar,^30^ 2021	India	77	Multicentre	30	30	Intensive care unit	At least 6 h per day	The proportion of patients engaged in awake prone positioning in both the intervention and control groups	5.5 h per day for up to 1 week	1 h per day for up to 1 week
Ehrmann,^7^ 2021	France	68	Multicentre	200	202	Intensive care unit	At least 16 h per day, if possible	Endotracheal Intubation or death within four weeks of randomisation	2.0 [1.0 to 3.7] h per day for up to 2 weeks	0 h per day
Yarahmadi,^27^ 2023	Iran	58	Single-centre	41	41	Ward	1.5 h session followed by a total of 8 h during the day of hospitalisation	Peripheral oxygen saturation. respiratory rate (breaths min^−1^), heart rate (beats min^−1^), mean arterial pressure (mmHg), the severity of dyspnoea (Visual Analogue Scale) [time frame: these outcomes were collected immediately before the intervention, and after 30 min, 1, 1.5, and 2 h]	Not applicable	Not applicable
Hashemian,^28^ 2021	Iran	58	Single-centre	45	30	Intensive care unit	30 min every 4 h	Arterial partial pressure of oxygen to fraction of inspired oxygen ratio at the end of the last non-invasive ventilation or non-invasive ventilation + awake prone positioning session on the first intervention day	Not applicable	Not applicable
Rampon,^33^ 2022	Spain and the United States	57 & 40 respectively	Multicentre	159	134	Ward	1 to 2 h per session at any time of day for 12 h	Composite of respiratory deterioration (supplementary oxygen requirement increase) or intensive care unit transfer	71.4% of patients underwent awake prone positioning at least once; 35.7% of patients underwent awake prone positioning for at least 6 h at least once	59.4% of patients underwent awake prone positioning at least once, and 13% of patients underwent awake prone positioning for at least 6 h at least once
Ehrmann,^7^ 2021	Spain	57	Multicentre	17	13	Intensive care unit	At least 16 h per day, if possible	Endotracheal intubation or death within four weeks of randomisation	1.6 [1.1 to 2.3] h per day for up to 2 weeks	0 [0] h per day
Javed,^26^ 2023	Pakistan	55	Single-centre	36	36	Ward	8 h per day (with cycles greater than 30 min but less than 3 h) for 1 week	Death within the first week of hospitalisation, awake prone positioning period, and less than 90 days from admission	8 h per day for up to 1 week	0 h per day for up to 1 week
Ehrmann,^7^ 2021	The United States	40	Multicentre	112	110	Ward, high-density unit, and intensive care unit	At least 16 h per day, if possible	Endotracheal Intubation or death within 4 weeks of randomisation	2.5 [0.7 to 6.9] hours per day for up to 2 weeks	0 [0] h per day
Johnson,^31^ 2021	The United States	40	Single-centre	15	15	Ward	1 to 2 h or as long as possible every 4 h	Change in arterial partial pressure of oxygen to fraction of inspired oxygen ratio after 3 days	1.6 [0.2 to 3.1] h per 3 days	0 [0] h per 3 days
Taylor,^35^ 2021	The United States	40	Single-centre	28	13	Ward	As tolerated with the intent of undergoing awake prone positioning for a total of at least 48 h	1. Acceptability 2. Adoption 3. Appropriateness 4. Effectiveness 5. Equity 6. Feasibility 7. Fidelity 8. Penetration	Not applicable	Not applicable
Fralick,^9^ 2022	Canada and the United States	39 (Canada), 40 (United States)	Multicentre	126	122	Ward	Over 12 h per day (every 4 days for at least 2 h)	1. In-hospital all-cause mortality 2. Mechanical ventilation 3. Need for at least 60% supplementary oxygen for more than 1 day	6 [1.5 to 12.9] h per 3 days	0 [0 to 2] h per 3 days
Ehrmann,^7^ 2021	Canada	39	Multicentre	7	6	Ward, high-density unit, and intensive care unit	At least 16 h per day, if possible	Endotracheal Intubation or death within 4 weeks of randomisation	2.4 [1.7 to 3.0] h per day for up to 2 weeks	0 [0] h per day
Kharat,^32^ 2021	Switzerland	34	Single-centre	10	17	Ward	12 h per day	1. Oxygen needs assessed by nasal cannula 2. Oxygen flow after 1 day	4.9 [2.3 to 7.5] h per day for up to 1 day	0 [0] h per 3 days
Rosén,^34^ 2021	Sweden	31	Multicentre	36	39	Ward and intensive care unit	12 h per day	Endotracheal intubation within 30 days of enrolment	9.0 [4.4 to 10.6] h per day for up to 3 days	3.4 [4.4 to 10.6] h per day for up to 3 days
Ehrmann,^7^ 2021	Ireland	28	Multicentre	12	12	Ward, high-density unit, and intensive care unit	At least 16 h per day, if possible	Endotracheal intubation or death within 4 weeks of randomisation	3.1 [2.1 to 3.9] h per day for up to 2 weeks	0 [0] h per day

### Risk of bias assessment

Thirteen of the included studies were deemed to have a low risk of bias,^7–9,18–21,26–28,30,33,35^ three had unclear risk of bias^30,31,32^ and one had a high risk due to bias in allocation concealment^34^ (Supplemental Data Figures 1 and 2).

### Primary outcomes

#### Endotracheal intubation

**Fig. 3:**
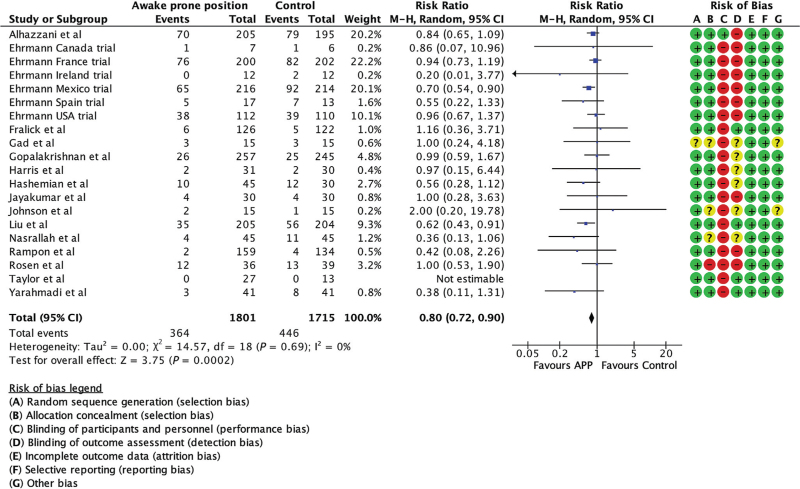
Forest plot comparing intubation in awake prone position and usual care groups.

**Fig. 4:**
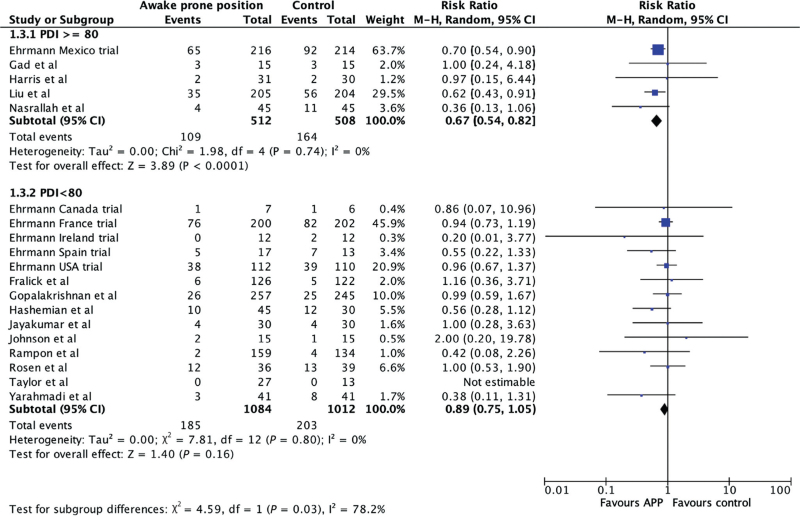
Forest plot risk of intubation with awake prone positioning versus no awake prone positioning, stratified by the Power Distance Index in the country where the trial was conducted.

APP, awake prone positioning; PDI, Power Distance Index.

Of the 22 trials, 20 trials including 3516 patients reported intubation as an outcome^7–9,18–21,27–31,33–35^ two trials did not.^26,32^ The pooled analysis of 20 trials showed a statistically significant benefit for avoiding intubation in the APP group compared to standard care (RR 0.80, 95% CI, 0.72 to 0.90, *I*^2^ = 0%, Fig. 3). When stratified by PDI (≥80 and <80, Fig. 4), the test for subgroup differences was significant (*P* = 0.03). The distribution of trials was unequal between the two groups with PDIs at least 80 having fewer participants (5 trials, 512 participants) than PDIs less than 80 (14 trials, 2096 participants). The effect estimate for PDIs at least 80 favoured APP with reduced endotracheal intubation (RR 0.67, 95% CI, 0.54 to 0.82). The effect was not statistically significant in the PDI less than 80 group (RR 0.89, 95% CI, 0.75 to 1.05). There was no heterogeneity within these strata. The results were consistent with exclusion of RCTs with high risk of bias^34^ and with the exclusion of unclear and high risk of bias^29,31,34^ (Supplemental Data Figures 3 and 4). The subgroup analysis by duration of APP showed a statistically significant sub-group difference (*P* = 0.06, Supplemental Data Figure 5). The ≥ 8 h per day APP duration group showed a significant reduction in intubation (RR 0.70, 95% CI, 0.57 to 0.86, *I*^2^ = 0%) compared to the group with APP duration less than 8 h per day (RR 0.89, 95% CI 0.77 to 1.04, *I*^2^ = 0%).

#### Mortality

**Fig. 5:**
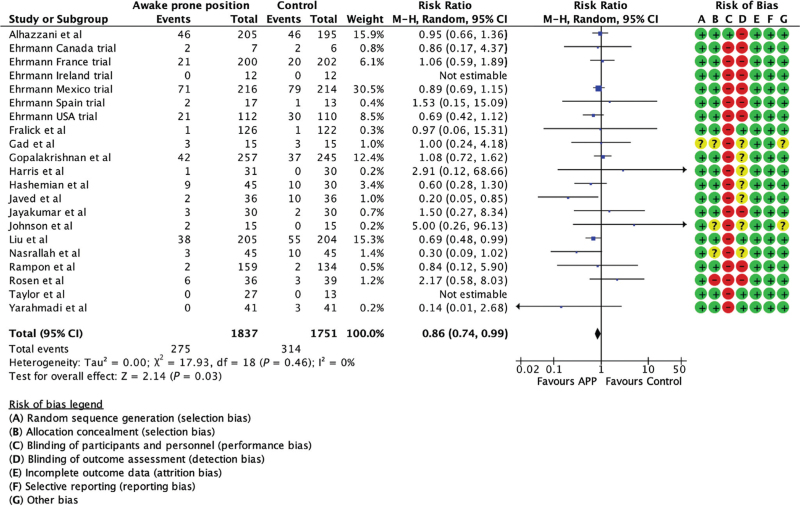
Forest plot comparing mortality in the awake prone positioning and usual care groups.

The pooled analysis of 21 trials (3588 patients)^7–9,18–21,26–31,33–35^ that reported mortality as outcome showed a statistically significant difference in mortality between the APP and standard care groups (RR 0.86, 95% CI 0.74 to 0.99, *I*^2^ = 0%, Fig. 5). One trial did not report mortality as outcome.^32^ However, the analysis did not show any statistically significant benefits when stratified by PDI (Supplemental Figure 6).

### Secondary outcomes

Fourteen trials reported the duration of APP.^7–9,20,21,30–32,34^ The *χ*^2^ test revealed a statistically significant association between PDI (<80 and ≥80) and APP duration (<8 and ≥8 h), *P* = 0.04 (Supplemental Data Table 3). Table 2 and Supplemental Data Figure 7 illustrate this relationship, showing that patients in higher PDI countries benefit more from APP by adopting it for a longer period.

**Table 2:** Awake prone positioning duration, PDI and outcome of the study

Trial	Duration of APP in h day^−1^ (median or mean)	Result^a^	Country	PDI	Number of APP patients
Harris^20^	6.2	Negative	Qatar	93	31
Ehrmann^7^	8.6	Positive	Mexico	81	216
Liu^21^	12	Positive	China	80	205
Gad^29^	Not mentioned	Negative	Egypt	80	15
Nasrallah^19^	Not mentioned	Positive	Egypt	80	45
Gopalakrishanan^18^	Not mentioned	Negative	India	77	257
Jayakumar^30^	4	Negative	India	77	30
Ehrmann^7^	2	Negative	France	68	200
Hashemian^28^	Not mentioned	Negative	Iran	58	45
Yarahmadi^27^	Not mentioned	Positive	Iran	58	41
Ehrmann^7^	1.6	Negative	Spain	57	17
Javed^26^	Not mentioned	Positive	Pakistan	55	36
Ehrmann^7^	2.5	Negative	USA	40	112
Fralick^9^	2	Negative	USA	40	122
Johnson^31^	0.5	Negative	USA	40	15
Taylor^35^	Not mentioned	Negative	USA	40	27
Ehrmann^7^	2.4	Negative	Canada	39	7
Kharat^32^	4.9	Negative	Switzerland	34	10
Rosen^34^	9	Negative	Sweden	31	36
Ehrmann^7^	3.1	Negative	Ireland	28	12
Rampon^33^	Not mentioned	Negative	Spain and USA	57, 40	159
Alhazzani^8^	6	Negative	USA, Canada, Kuwait, Saudi	40, 39, 90, 90	205

APP, awake prone positioning; PDI, Power Distance Index.

a Study was classified as positive (statistically significant benefit found) and negative (no statistically significant benefit found).

Eight studies (2348 patients) studied escalation of respiratory support.^7,9,18,20,30–33^ The difference was not statistically significant (RR 1.02, 95% CI, 0.81 to 1.30, *I*^2^ = 39%, Supplemental Data Figure 8). Seven studies with 575 patients reported the need for ICU admission.^7,20,31–35^ The pooled analysis was comparable in both the APP and standard care groups (RR 0.90, 95% CI, 0.65 to 1.24, *I*^2^ = 25%, Supplemental Data Figure 9).

Changes in SpO_2_ : FiO_2_ ratio pre-APP and post-APP were reported by five studies,^8,9,32,34,36^ hospital length of stay was reported by nine^7,18,20,27,29,31,32,34,35^ and the incidence of adverse events was reported by eight.^7–9,19–21,30,34^ Due to the presence of high heterogeneity, no analysis was done for these outcomes. The adverse events reported most frequently were pain or discomfort, vomiting and central line dislodgement (Supplemental Data Table 3).

#### Sensitivity analysis

Sensitivity analysis was carried out by excluding trials with a high risk of bias (75 patients)^34^ and trials with a high and unclear risk of bias (135 patients).^29,31,34^ The analyses showed results consistent with the primary analysis for both endotracheal intubation and mortality (Supplemental Data Figures 3, 4, 10 and 11).

#### Certainty of evidence

Certainty of evidence was assessed using the Grading of Recommendations, Assessment, Development, and Evaluation (GRADE) approach for all outcomes and is shown in Supplemental Data Table 4.

#### Publication bias

Publication bias assessed using funnel plots for primary outcomes, intubation and mortality, did not show a bias (Supplemental Data Figures 12 and13). The number of studies was insufficient to assess publication bias for secondary outcomes.

## Discussion

This systematic review and meta-analysis, the largest such study to date, included 22 RCTs to November 2024 with 3615 individuals randomised to either APP or standard care. It found that APP, a safe nonpharmacologic intervention in adults in hospital with COVID-19 ARDS, reduced the relative risk of endotracheal intubation by 20% (95% CI, 10 to 28%, Fig. 2). The subgroup differences in the analysis of effect for PDIs was statistically significant. The benefit was present in nations with a high PDI (33% reduction, 95% CI, 18 to 46%), but not in nations with a low PDI (11% reduction, 95% CI, 25% reduction to 5% increase, Fig. 3). Pooled analysis demonstrated a statistically significant reduction in mortality by 14% (95% CI, 1 to 26%). Point estimates were lower among countries with a high PDI versus those with a low PDI (Fig. 4). We also found that the intubation rates were lower when APP duration more than or equal to 8 h per day and the countries with higher PDI had a longer duration of APP. No adverse events were observed.

As we had hypothesised, heterogeneity of APP treatment effect correlates with national PDI, but the RCTs were not evenly distributed between low and high PDI groups. Limited resources and funds in high PDI countries such as Mexico and Egypt might have contributed to fewer RCTS form these countries. The large COVI-PRONE trial, which included both countries with high PDI, including Kuwait and Saudi Arabia with 211 participants, and countries with low PDI, including Canada and the United States with 189 participants, did not report results by country and was excluded from the analysis.^8^ On average, individuals in RCTs conducted in countries with higher PDI are more deferential to instructions to adopt APP.^15,16^ Higher median duration of APP in the intervention group was associated with a high PDI (≥80). This is because individuals in high PDI countries tend to obey instructions more strictly, unlike people in countries with low PDIs who distribute power and are less willing to comply. Stepwise multiple regression analysis for the effect of PDI on COVID-19 mortality in 31 very highly developed European countries revealed a significant negative correlation.^37^ Regarding the spread of COVID-19, Huang *et al.* ^16^ reported it to be faster in countries with low PDI, as people were more suspicious of their leaders’ orders and lockdown rules. Adoption of anti-COVID-19 measures, such as masking, also saw a strong correlation with PDI in a study by Kamp *et al.* ^38^ with the principal component analysis pinpointing this dimension, among Hofstede's six dimensions, as the strongest predictor of adoption. Human behaviour and culture ultimately guide acceptance and implementation. The significance of this is highlighted in our analysis, which demonstrated a reduction in intubation rates with APP durations of at least 8 h, consistent with findings from a previous meta-analysis that included both clinical trials and observational studies.^11^ This is further supported by observations from the COVID PRONE RCT conducted in Canada and the United States but terminated due to futility, where participants maintained APP for an average of 6 h over a 72 h period, and falling short of the 8 h daily target.^9^ Similarly, in the COVI-PRONE RCT, participants maintained APP for an average of only 4.8 h per day with no observed reduction in intubation rates.^8^ In addition to cultural factors, the scarcity of resources and the cost of treatment in countries with high PDI may have further contributed to greater treatment adherence.

There is a strong physiological rationale for the potential benefits of APP in countries with low PDI if both adoption and duration are increased. This includes a decrease in ventilation–perfusion mismatch and lung injury, along with a more even distribution of stress that could alleviate self-induced lung injury from high respiratory drive.^39,40^ This is reflected in the consistent association of APP with reduced respiratory rates.^4,5,7^ Thus, the emphasis should be on quantitative studies using real-world evidence to compare whether APP for more than 8 h in US hospitals is associated with benefit.

**Fig. 6:**
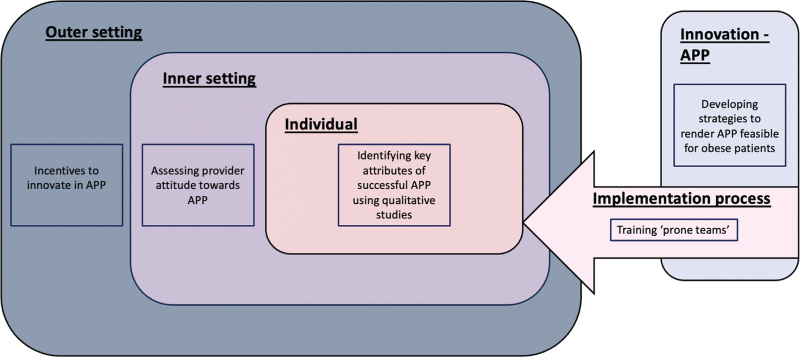
Implementation strategies for awake prone positioning using the Consolidated Framework for Implementation Research framework.

COVID-PRONE used various strategies to enhance APP duration including in-person directions, follow-up reminder phone calls, follow-up in-person visits, reminders for nurses and electronic medical record order sets but was terminated due to futility.^9^ In contrast, the RCT of Liu *et al.* reported a median APP duration of 12 h per day. Their approach was physician-driven, with clinicians trained to assist patients in finding the most comfortable prone position. Additionally, they incorporated music as a distraction and administered analgesics and sedatives when necessary to enhance patient comfort.^21^ Qualitative studies are essential to identify obstacles to implementation, as awareness and familiarity with APP do not seem to increase its adoption. Practice inertia, low self-efficacy, and low outcome expectancy may mediate the lack of implementation. An evidence-based framework, such as the Consolidated Framework for Implementation Research (CFIR), must be used to develop interventions to increase adoption. CFIR categorises the barriers to implementation into five domains.^41^ In 2016, it was employed to implement the use of patient-preferred music medicine, an evidence-based practice to decrease acute postoperative pain. A list of effective nonpharmacologic treatments that include auricular acutherapy and aromatherapy has been expanded.^42^ It is proposed that the organisation of efforts to increase APP should be based on the consideration of the five domains of CFIR: the outer setting (consideration of external influences, such as incentives and reimbursement for current procedural terminology codes, to motivate hospitals to innovate in APP), the inner setting (assessing the internal organisational environment for APP implementation involves evaluating provider attitudes, self-efficacy and exploring the effectiveness of proning teams versus training all providers), the characteristics of individuals (understanding the attributes linked to successful APP adoption, and examining qualitative studies to identify themes regarding APP implementation from the perspectives of providers and patients), the process (forming protocols to adopt APP) and the intervention (making adoption of APP easier for obese patients) (Fig. 6).^43^ Behavioural nudges, taking into account local culture (emphasising the global burden rather than imposing authority), may increase APP adherence and use. ‘Culture eats evidence for breakfast’ is a familiar quote, and patients are far more likely, on average, to comply when the room is set-up to promote APP as the default position (e.g. the bed is turned to see the television in the prone position).

### Strengths and limitations

This meta-analysis was thorough and current, complying with quality standards and presented an exhaustive examination of important clinical outcomes. To present the most reliable clinical evidence available, only RCTs were included.

This study is not without limitations. Inevitably, all the included RCTs were conducted without blinding, which has the potential to introduce bias. The RCTs included patients with ARDS/AHRF due to COVID-19 but, given the underlying pathophysiology, non-COVID-related ARDS is also likely to benefit from APP. In addition, the differences in treatment protocols, disease severity and oxygen requirements across trials might have influenced the results.

In conclusion, this review found that APP, an inexpensive intervention for ARDS, significantly reduced intubation in RCTs conducted in countries with high PDIs. Conversely, suboptimal adherence to APP in countries with low PDIs suggests potential enhancement through collaboration with researchers and the application of implementation frameworks. The diminished reliance on mechanical ventilation not only stands to reduce cost but also alleviate the burden on healthcare providers. Funding aimed at further investigations that encompass these outcomes, alongside traditional clinical metrics, is warranted, increasing the likelihood of widespread adoption of these findings.

## Supplementary Material

Supplemental Digital Content

## References

[1] Fan E Del Sorbo L Goligher EC . An official ATS/ESICM/SCCM clinical practice guideline: mechanical ventilation in adult patients with acute respiratory distress syndrome. *Am J Respir Crit Care Med* 2017; 195:1253–1263. 28459336 10.1164/rccm.201703-0548ST

[2] Guérin C Reignier J Richard JC . Prone positioning in severe acute respiratory distress syndrome. *N Engl J Med* 2013; 368:2159–2168. 23688302 10.1056/NEJMoa1214103

[3] Venus K Munshi L Fralick M . Prone positioning for patients with hypoxic respiratory failure related to COVID-19. *CMAJ* 2020; 192:E1532–E1537. 33177104 10.1503/cmaj.201201 PMC7721267

[4] Li J Luo J Pavlov I . Awake Prone Positioning Meta-Analysis Group Awake prone positioning for nonintubated patients with COVID-19-related acute hypoxaemic respiratory failure: a systematic review and meta-analysis. *Lancet Respir Med* 2022; 10:573–583. 35305308 10.1016/S2213-2600(22)00043-1 PMC8926412

[5] Weatherald J Parhar KKS Al Duhailib Z . Efficacy of awake prone positioning in patients with covid-19 related hypoxemic respiratory failure: systematic review and meta-analysis of randomized trials. *BMJ* 2022; 379:e071966. 36740866 10.1136/bmj-2022-071966 PMC9727649

[6] Graziani M Rigutini AG Bartolini D . Awake prone positioning for patients with COVID-19-related respiratory failure: a systematic review and meta-analysis. *Intern Emerg Med* 2023; 19:37796372. 10.1007/s11739-023-03434-1 PMC10827908 37796372

[7] Ehrmann S Li J Ibarra-Estrada M . Awake Prone Positioning Meta-Trial Group Awake prone positioning for COVID-19 acute hypoxaemic respiratory failure: a randomised, controlled, multinational, open-label meta-trial. *Lancet Respir Med* 2021; 9:1387–1395. 34425070 10.1016/S2213-2600(21)00356-8 PMC8378833

[8] Alhazzani W Parhar KKS Weatherald J . COVI-PRONE Trial Investigators and the Saudi Critical Care Trials Group Effect of awake prone positioning on endotracheal intubation in patients with COVID-19 and acute respiratory failure: a randomized clinical trial. *JAMA* 2022; 327:2104–2113. 35569448 10.1001/jama.2022.7993 PMC9108999

[9] Fralick M Colacci M Munshi L . COVID Prone Study Investigators Prone positioning of patients with moderate hypoxaemia due to COVID-19: multicentre pragmatic randomised trial (COVID-PRONE). *BMJ* 2022; 376:e068585. 35321918 10.1136/bmj-2021-068585 PMC8941343

[10] Qin S Chang W Peng F . Awake prone position in COVID-19-related acute respiratory failure: a meta-analysis of randomized controlled trials. *BMC Pulm Med* 2023; 23:145. 37101160 10.1186/s12890-023-02442-3 PMC10131466

[11] Vásquez-Tirado GA Meregildo-Rodríguez ED Asmat-Rubio MG . Conscious prone positioning in nonintubated COVID-19 patients with acute respiratory distress syndrome: systematic review and meta-analysis. *Crit Care Sci* 2024; 36:e20240176en. 10.62675/2965-2774.20240176-en PMC11098076 38597483

[12] Raghunathan K . Checklists, safety, my culture and me. *BMJ Qual Saf* 2012; 21:617–620. 10.1136/bmjqs-2011-000608 22491530

[13] Hofstede GH Hofstede GJ Minkov M . Cultures and organizations: software of the mind. Chicago: McGraw-Hill; 2010.

[14] Hofstede G. Clearly cultural: making sense of cross cultural communication. Available at: https://clearlycultural.com. [Accessed 7 December 2024]

[15] Kumar R . Impact of societal culture on covid-19 morbidity and mortality across countries. *J Cross-Cultural Psychol* 2021; 52:643–662.

[16] Huang X Gupta V Feng C . How national culture influences the speed of COVID-19 spread: three cross-cultural studies. *Cross Cult Res* 2023; 57:193–238. 38603334 10.1177/10693971221141478 PMC9703026

[17] Cao W He N Luo Y . Awake prone positioning for nonintubated patients with COVID-19-related acute hypoxic respiratory failure: a systematic review based on eight high-quality randomized controlled trials. *BMC Infect Dis* 2023; 23:415. 37337193 10.1186/s12879-023-08393-8 PMC10278266

[18] Gopalakrishnan M Khichar S Saurabh S . Effectiveness of early awake self proning strategy in nonintubated patients with COVID-19 hypoxemia: an open-labelled randomized clinical trial from Jodhpur, India. *Monaldi Arch Chest Dis* 2022; 93: 10.4081/monaldi.2022.2431 36524853

[19] Nasrallah BZN Mahmoud MS Abdallah ElGendy HM . Patients self-proning with high-flow nasal cannula improves oxygenation in ARDS patients: a randomized clinical trial. *Anaesth Pain Intensive Care* 2023; 27:351−355.

[20] Harris TRE Bhutta ZA Qureshi I . A randomised clinical trial of awake prone positioning in COVID-19 suspects with acute hypoxemic respiratory failure. *Contemp Clin Trials Commun* 2024; 39:101295. 38689829 10.1016/j.conctc.2024.101295 PMC11059337

[21] Liu L Sun Q Zhao H . Chi-ARDS Net (Chinese ARDS Research Network) Prolonged vs shorter awake prone positioning for COVID-19 patients with acute respiratory failure: a multicenter, randomised controlled trial. *Intensive Care Med* 2024; 50:1298–1309. 39088076 10.1007/s00134-024-07545-x PMC11306533

[22] Page MJ Moher D Bossuyt PM . PRISMA 2020 explanation and elaboration: updated guidance and exemplars for reporting systematic reviews. *BMJ* 2021; 372:n160. 33781993 10.1136/bmj.n160 PMC8005925

[23] *Cochrane Handbook for Systematic Reviews of Interventions* version 6.3 (updated August 2022): Cochrane; 2022. Available at: www.training.cochrane.org/handbook [Accessed 30 June 2023]

[24] Richardson M Garner P Donegan S . Interpretation of subgroup analyses in systematic reviews: a tutorial. *Clin Epidemiol Global Health* 2019; 7:192–198.

[25] Guyatt G Oxman AD Akl EA . GRADE guidelines: 1. Introduction-GRADE evidence profiles and summary of findings tables. *J Clin Epidemiol* 2011; 64:383–394. 21195583 10.1016/j.jclinepi.2010.04.026

[26] Javed H Qayyum F Khan A . Effect of eight hours per day of intermittent self prone positioning for seven days on the severity of Covid-19 pneumonia/acute respiratory distress syndrome. *J Ayub Med Coll Abbottabad* 2023; 35:68–75. 36849380 10.55519/JAMC-01-11069

[27] Yarahmadi S Ebrahimzadeh F Mohamadipour F . Effect of prone position on clinical outcomes of nonintubated patients with COVID-19: a randomised clinical trial. *Collegian* 2023; 30:449–456. 36591534 10.1016/j.colegn.2022.12.005 PMC9792421

[28] Hashemian SM Jamaati H Malekmohammad M . Efficacy of early prone positioning combined with noninvasive ventilation in COVID-19. *Tanaffos* 2021; 20:82–85. 34976078 PMC8710224

[29] Gad GS . Awake prone positioning versus non invasive ventilation for COVID-19 patients with acute hypoxemic respiratory failure. *Egypt J Anaesth* 2021; 37:85–90.

[30] Jayakumar D Ramachandran Dnb P Rabindrarajan Dnb E . Standard care versus awake prone position in adult nonintubated patients with acute hypoxemic respiratory failure secondary to COVID-19 infection-a multicenter feasibility randomized controlled trial. *J Intensive Care Med* 2021; 36:918–924. 33949237 10.1177/08850666211014480 PMC8107489

[31] Johnson SA Horton DJ Fuller MJ . Patient-directed prone positioning in awake patients with COVID-19 requiring hospitalization (PAPR). *Ann Am Thorac Soc* 2021; 18:1424–1426. 33596394 10.1513/AnnalsATS.202011-1466RL PMC8513661

[32] Kharat A Dupuis-Lozeron E Cantero C . Self-proning in COVID-19 patients on low-flow oxygen therapy: a cluster randomised controlled trial. *ERJ Open Res* 2021; 7:692–2020. 10.1183/23120541.00692-2020 PMC7869594 33718487

[33] Rampon G Jia S Agrawal R . Smartphone-guided self-prone positioning vs usual care in nonintubated hospital ward patients with COVID-19: a pragmatic randomized clinical trial. *Chest* 2022; 162:782–791. 35597286 10.1016/j.chest.2022.05.009 PMC9116967

[34] Rosén J von Oelreich E Fors D . PROFLO Study Group Awake prone positioning in patients with hypoxemic respiratory failure due to COVID-19: the PROFLO multicenter randomized clinical trial. *Crit Care* 2021; 25:209. 34127046 10.1186/s13054-021-03602-9 PMC8200797

[35] Taylor SP Bundy H Smith WM . Awake prone positioning strategy for nonintubated hypoxic patients with COVID-19: a pilot trial with embedded implementation evaluation. *Ann Am Thorac Soc* 2021; 18:1360–1368. 33356977 10.1513/AnnalsATS.202009-1164OC PMC8513648

[36] Ibarra-Estrada M Li J Pavlov I . Factors for success of awake prone positioning in patients with COVID-19-induced acute hypoxemic respiratory failure: analysis of a randomized controlled trial. *Crit Care* 2022; 26:84. 35346319 10.1186/s13054-022-03950-0 PMC8958810

[37] Gokmen Y Baskici C Ercil Y . The impact of national culture on the increase of COVID-19: a cross-country analysis of European countries. *Int J Intercultural Relat* 2021; 81:1–8. 10.1016/j.ijintrel.2020.12.006 PMC7833793 33518841

[38] Kamp B Gibaja JJ San Martin J . Adoption of measures to mitigate the impact of COVID-19: in search of a Hofstedian explanation for patterns among individual countries and country clusters. *saf Sci* 2023; 157:105902. 36061517 10.1016/j.ssci.2022.105902 PMC9420697

[39] Edgcombe H Carter K Yarrow S . Anaesthesia in the prone position. *Br J Anaesth* 2008; 100:165–183. 18211991 10.1093/bja/aem380

[40] Chen L Zhang Y Li Y . The application of awake-prone positioning among nonintubated patients with COVID-19-related ARDS: a narrative review. *Front Med* 2022; 9:817689. 10.3389/fmed.2022.817689 PMC8858818 35198575

[41] Damschroder LJ Reardon CM Widerquist MAO . The updated Consolidated Framework for Implementation Research based on user feedback. *Implementation Sci* 2022; 17:75. 10.1186/s13012-022-01245-0 PMC9617234 36309746

[42] Carter JE Pyati S Kanach FA . Implementation of perioperative music using the consolidated framework for implementation research. *Anesth Analg* 2018; 127:623–631. 29905616 10.1213/ANE.0000000000003565

[43] Zaretsky J Corcoran JR Savage E . Increasing rates of prone positioning in acute care patients with COVID-19. *Jt Comm J Qual Patient Saf* 2022; 48:53–60. 34848158 10.1016/j.jcjq.2021.09.005 PMC8444473

