# Supplementary material for: The effect of culture on the benefits of awake prone positioning for adults with COVID-19 acute respiratory distress syndrome: A systematic review and meta-analysis
Source: Eur J Anaesthesiol Intensive Care. 2025 Feb 5;4(2):e0068. doi: 10.1097/EA9.0000000000000068 (PMC11977753; doi:10.1097/EA9.0000000000000068)
Supplement: Supplemental Digital Content [file ejaic-4-e0068-s001.docx]

**A Systematic Review and Meta-Analysis of Randomised-Controlled Trials on Awake-Prone Positioning among Adults with Coronavirus Disease 2019 Acute Respiratory Distress Syndrome: Heterogeneity of Treatment Effects based on Power Distance Index**

Supplemental Material

Contents

Supplemental material - Search strategy 3

Supplemental Table 1 4

Supplemental Table 2 12 Figure 1 18

Figure 2 19

Figure 3 20

Figure 4 21

Figure 5 22

Figure 6 23

Figure 7 24

Figure 8 25

Figure 9 26

Figure 10 27

Figure 11 28

Figure 12 29

Figure 13 30

Figure 14 31

Supplemental Table 3 32

Supplemental Table 4 33

**Supplemental material - Search Strategy**

Database: Interface – EBSCOhost, Database – CINAHL Complete. Limiters – English Language

| # | Query | Results |
| --- | --- | --- |
| S1 | Acute Respiratory Distress Syndrome | 4183 |
| S2 | Acute Hypoxaemic Respiratory Failure | 139 |
| S3 | Self Prone | 36 |
| S4 | Prone Position | 1675 |
| S5 | Awake Prone Position | 5 |
| S6 | S1 OR S2 | 4291 |
| S7 | (S1 OR S2) AND (S3 OR S4 OR S5) | 198 |

Database: Scopus

( ( TITLE-ABS-KEY ( acute  AND respiratory  AND distress  AND syndrome )  OR  TITLE-ABS KEY ( acute  AND hypoxemic  AND respiratory  AND failure ) ) )  AND  ( ( TITLE-ABS-KEY ( self  AND prone )  OR  TITLE-ABS-KEY ( prone  AND position )  OR  TITLE-ABS-KEY ( awake  AND prone  AND position ) ) )  AND  ( LIMIT-TO ( LANGUAGE ,  "English" ) ) – 1761 documents

Database: Embase

| # | Query | Results |
| --- | --- | --- |
| 1 | Acute Respiratory Distress Syndrome | 29440 |
| 2 | Acute AND ‘Hypoxemic Respiratory Failure’ AND Respiratory AND Failure | 1407 |
| 3 | Self AND Prone | 4348 |
| 4 | Awake AND Prone AND Position | 254 |
| 5 | ‘Prone Position’ | 7640 |
| 6 | #1 OR #2 | 26408 |
| 7 | #3 OR #4 OR #5 | 11843 |
| 8 | #6 AND #7 | 934 |

Database: The Cochrane Library

| # | Query | Results |
| --- | --- | --- |
| 1 | (“Acute Respiratory Distress Syndrome”) OR (“Acute Hypoxemic Respiratory Failure”) | 2733 |
| 2 | (Self Prone) OR (Prone Position) OR (Awake Prone Position) | 2504 |
| 3 | #1 AND #2 | 241 |

Supplemental Table 1: Inclusion Criteria, Oxygen Delivery Modalities, Intervention, Control Group Details, and Follow-up Duration of Included Randomised-Control Trials

| **1^st^ Author (Nation(s))** | **Inclusion Criteria** | **Oxygen Delivery Modalities** | **Intervention Details** | **Control Details** | **Follow-Up Duration** |
| --- | --- | --- | --- | --- | --- |
| Harris (Qatar) | 1. Adults at least eighteen years of age with suspected or confirmed Coronavirus disease 2019 pneumonia 2. Patients who required at least five liters per minute of supplemental oxygen via Face Mask or NFNC/Non-Invasive ventilation with a Fraction of Inspired Oxygen at least 0.4 and or a Positive End Expiratory Pressure of at least five centimeters of water to achieve a Peripheral Oxygen Saturation of at least ninety-four percent with pulse oximetry 3. Required oxygen therapy to be initiated within one day of hospitalisation | 1. Simple Supplementary Oxygen 2. Non-Invasive Ventilation | 1. Awake-Prone Positioning for as long as possible up to three per session with three sessions per day | 1. Preferred/comfortable position | Thirty days |
| Alhazzani (Canada, Kuwait, Saudi Arabia, and the United States) | 1. At least eighteen years old 2. Suspected or Confirmed Coronavirus Disease 2019 3. Hypoxaemia Requiring Oxygen supplementation with a Fraction of Inspired Oxygen greater than or equal to 0.4 or at least five liters per minute on Nasal Cannula 4. Bilateral or Unilateral Chest Infiltrates on Chest X-Ray as interpreted by the Treating Team 5. Admitted to Intensive or Acute Care Unit where Hemodynamic and Respiratory Monitoring is feasible | 1. High-Flow Oxygen 2. Low-Flow Oxygen 3. Noninvasive Positive Pressure Ventilation | 1. Awake-Prone Positioning until forty percent relative Fraction of Inspired Oxygen requirement improvement from baseline for one day, Endotracheal Intubation, or discharge from Intensive or Acute Care Unit | 1. Usual Care | Thirty days |
| Ehrmann (Mexico) | 1. At least eighteen years old with Reverse Transcriptase Polymerase Chain Reaction confirmed Coronavirus Disease 2019 and Respiratory Distress (regardless of Acute Respiratory Distress Syndrome Berlin criteria) 2. Requirement of at least thirty percent Fraction of Inspired Oxygen through High Flow Nasal Cannula to maintain a Peripheral (Capillary) Oxygen Saturation greater than or equal to ninety percent 3. Written informed consent | 1. High Flow Nasal Cannula at forty liters per minute and thirty-seven degrees Celsius according to patient comfort and tolerance 2. Fraction of Inspired Oxygen titrated to a Peripheral (Capillary) Oxygen Saturation of ninety-two to ninety-five percent 3. High Flow Nasal Cannula withdrawn when the Fraction of Inspired Oxygen is less than or equal to 0.4 | 1. Patients underwent Awake-Prone Positioning for the maximum amount of time tolerable 2. Awake-Prone Positioning was stopped upon High Flow Nasal Cannula weaning, discharge, Endotracheal Intubation, or death 3. Endotracheal Intubation initiated upon worsening respiratory failure (Respiratory Rate less than forty breaths per minute, respiratory muscle weakness, respiratory acidosis with a pH less than 7.25, excessive tracheal secretions, Peripheral Oxygen Saturation less than ninety percent even if the Fraction of Inspired Oxygen is greater than or equal to 0.8, hemodynamically unstable, or worsening mentation) | 1. High Flow Nasal Cannula with the intent of ninety to ninety-five percent 2. Usual Care | Twenty-eight days |
| Liu (China) | 1. Age eighteen to eighty five 2. Confirmed Coronavirus Disease 2019 Pneumonia 3. Not Intubated 4. Peripheral Oxygen Saturation less than or equal to ninety three percent with ambient air at rest or a Partial Pressure of Oxygen to Fraction of Inspired Oxygen ratio less than or equal to three hundred millimeters of mercury | 1. Standard Oxygen 2. High-Flow Nasal Oxygen 3. Mask Noninvasive Ventilation | 1. Lie Prone under caregiver supervision (to ensure that patients were on their chests rather than their sides) 2. Lie Prone for as long as possible and as close as possible to at least twelve hours per day for one week 3. Fraction of Inspired Oxygen titrated to maintain a Peripheral Oxygen Saturation greater than ninety percent | 1. Patients could decide whether or not to lie Prone dispute being told not | Twenty-eight days |
| Nasrallah (Egypt) | 1. Over eighteen years old 2. Body Mass Index less than thirty kilograms per square meter 3. Coronavirus Disease 2019 Positive with mild Acute Respiratory Distress Syndrome according to the Berlin Criteria (Bilateral Opacities on Chest Radiographs not fully explained by Effusions, Lobar/Lung collapse, or Nodules and Edema not of Cardiac origin or caused by Volume Overload) | 1. High Flow Nasal Cannula | 1. High Flow Nasal Cannula with a Target Peripheral Oxygen Saturation greater than or equal to ninety percent combined with Self-Proning | 1. High Flow Nasal Cannula with a target Peripheral Oxygen Saturation greater than or equal to ninety percent with a Fraction of Inspired Oxygen less than 0.6 | Twenty-eight days |
| Gad (Egypt) | 1. Hospitalized patients with a Positive Nasopharyngeal/Oropharyngeal Coronavirus Disease 2019 swab 2. Over eighteen years old 3. Arterial Oxygen Saturation less than ninety percent (five to ten liter per minute with a simple face mask) 4. Arterial Partial Pressure of Oxygen to Fraction of Inspired Oxygen ratio less than two hundred 5. Respiratory Rate greater than twenty-four breaths per minute 6. Bilateral Lung Infiltration in Chest Computed Tomography Scan not explained by Cardiac Failure 7. Ready to cooperate with Prone Positioning or Non-Invasive Ventilation | 1. High Flow Oxygen through a Non-Rebreather Mask at ten to fifteen liters per minute | 1. Awake-Prone Positioning sessions for one to two hours according to patient tolerability, three hours apart during waking hours | 1. Non-Invasive Ventilation sessions for one to two hours according to patient tolerability, three hours apart during waking hours | Three days |
| Gopalakrishnan (India) | 1. Peripheral Oxygen Saturation less than ninety-four percent on room air or requiring oxygen support by nasal prongs/facemask to maintain a Peripheral Oxygen Saturation greater than ninety-four percent 2. Able to communicate 3. Able to Self prone | 1. Nasal Prongs 2. Facemask | 1. Awake-Prone Positioning for thirty minutes to two hours followed by left-lateral positioning for thirty minutes, right-lateral positioning for thirty minutes, and supine or sitting for thirty minutes | 1. Intravenous dexamethasone and remdesivir 2. Subcutaneous Prophylactic Low Molecular Weight Heparin (if no contraindications) | Eight to two hundred ninety-four days |
| Jayakumar (India) | 1. At least eighteen years old 2. Requiring at least four liters per minute of supplemental oxygen to maintain a Peripheral Oxygen Saturation greater than or equal to ninety-two percent or an Arterial Partial Pressure of Oxygen to Fraction of Inspired Oxygen ratio greater than or equal to one hundred millimeters of mercury or less than or equal to three hundred millimeters of mercury with an Arterial Partial Pressure of Carbon Dioxide less than forty-five millimeters of mercury | 1. Face Mask 2. Non-Rebreather Mask 3. High Flow Nasal Cannula 4. Non-Invasive Ventilation 5. Nasal Prongs | 1. Patients in the intervention group were encouraged to undergo Awake-Prone Positioning 2. Oxygen flow rate, Fraction of Inspired Oxygen, and Positive End-Expiratory Pressure were optimized so that Peripheral Oxygen Saturation was greater than or equal to ninety-0two percent 3. Patients with face masks received oxygen at a rate less than or equal to ten liters per minute 4. Patients on High Flow Nasal Cannula received oxygen at the highest possible flow rate 5. Patients on blenders received oxygen at a flow rate less than or equal to sixty liters per minute 6. If the Fraction of Inspired Oxygen was less than or equal to 0.4, the flow rate was decreased by increments of ten liters per minute until a rate of twenty liters per minute was reached 7. If the Fraction of Inspired Oxygen was less than or equal to 0.3 with a flow rate of twenty liters per minute, High Flow Nasal Cannula was exchanged for either a face mask or nasal prongs 8. Oxygen was weaned if the flow rate was less than or equal to ten liters per minute 9. Patients undergoing Awake-Prone Positioning while receiving Non-Invasive Ventilation were provided with pillows | 1. Usual Care | Seven days |
| Ehrmann (France) | 1. Adults suffering from Coronavirus Disease 2019 pneumonia according to the diagnostic criteria in effect at the inclusion time or very strongly suspected 2. Patient treated by High-Flow Nasal Therapy 3. Moderate or severe Acute Respiratory Distress Syndrome: bilateral radiological opacities not entirely explained by atelectasis, nodules, or effusions; acute hypoxaemia with worsening within the previous week, not explained entirely by left ventricular failure; Arterial Partial Pressure of Oxygen to Fraction of Inspired Oxygen less than three hundred millimeters of mercury (or equivalent Peripheral Oxygen Saturation to Fraction of Inspired Oxygen) 4. Written informed consent | 1. High Flow Nasal Cannula adapted for a Peripheral Oxygen Saturation of ninety to ninety-five percent 2. An initial minimum flow rate of fifty liters per minute, unless poorly tolerated 3. High Flow Nasal Cannula 1^st^ weaned by decreasing the Fraction of Inspired Oxygen to 0.4, followed by flow rate reduction 4. Attempt made to switch to standard oxygen therapy at four to six liters per minute in clinically stable patients with a Fraction of Inspired Oxygen less than or equal to 0.4 and a flow rate less than or equal to thirty liters per minute | 1. Patients underwent Awake-Prone Positioning for the maximum tolerable amount of time 2. Awake-Prone Positioning was stopped upon High Flow Nasal Cannula weaning, discharge, Endotracheal Intubation, or death 3. Endotracheal Intubation initiated upon worsening respiratory failure (Respiratory Rate less than forty breaths per minute, respiratory muscle weakness, respiratory acidosis with a pH less than 7.25, excessive tracheal secretions, Peripheral Oxygen Saturation less than ninety percent even if the Fraction of Inspired Oxygen is greater than or equal to 0.8, hemodynamically unstable, or worsening mentation) | 1. High Flow Nasal Cannula with the intent of achieving a Peripheral Oxygenation Saturation of ninety to ninety-five percent 2. Usual Care | Twenty-eight days |
| Yarahmadi (Iran) | 1. Coronavirus Disease 2019 positive based on clinical symptoms and signs, computed tomography chest findings compatible with the COronavirus Disease 2019 pneumonia pattern, and a polymerase chain reaction test 2. Age thirty-five to seventy 3. Lack of supportive ventilation 4. Lack of chronic obstructive pulmonary disease or asthma 5. Lack of orthopaedic and spine disorders 6. No history of thoracic surgery during the last six months | 1. Twenty-one to sixty percent Fraction of Inspired Oxygen using a simple mask | 1. Patients were asked to lie comfortably in a Prone Position for an hour and a half and then return to a supine position 2. At the end of the hour-and-a-half period, the participants were asked to intermittently stay in a Prone Position for a total of eight hours during the day of hospitalisation | 1. Patients were asked to lie comfortably in a supine position at a thirty-degree angle for an hour and a half 2. At the end of the hour and a half, participants were asked to remain in their usual positions (other than the Prone) during the hospital stay | Patients were followed up until hospital discharge or occurrence of Endotracheal Intubation, or death |
| Hashemian (Iran) | 1. Coronavirus Disease 2019 patients 2. Body Mass Index greater than eighteen kilograms per square meter but less than thirty kilograms per square meter | 1. Non-Invasive Ventilation (Continuous Positive Airway Pressure) or BiPAP Bilevel Positive Airway Pressure Spontaneous/Timed mode via total face mask) 2. Non-Invasive Ventilation (Continuous Positive Airway Pressure or Bilevel Positive Airway Pressure Spontaneous/Timed mode Helmet masks) | 1. Patients undergoing Awake-Prone Positioning underwent Endotracheal Intubation if the Respiratory Rate was less than forty breaths per minute, experienced syncope or hemodynamic instability, or Partial Pressure of Carbon Dioxide less than fifty millimeters of mercury | 1. Usual Care 2. Non-Invasive Ventilation | One day |
| Rampon (Spain and the United States) | 1. At least eighteen years old 2. Admission to Ward or planned admission to Ward from ED within the previous two days 3. Confirmed Coronavirus Disease 2019 or under evaluation for Coronavirus Disease 2019 4. Access to a functioning smartphone that can connect to the internet and receive text messages in the hospital room 5. English or Spanish-speaking 6. Self-identified ability to read simple instructions and answer simple written questions | 1. Nasal Cannula 2. Mask 3. High Flow Nasal Cannula | 1. Patients in the intervention group received a link via text message to the Qualtrics intervention website with instructions regarding Awake-Prone Positioning | 1. Patients in the control group received a link via text message to the Qualtrics intervention website with instructions regarding usual care | Fourteen days |
| Ehrmann (Spain) | 1. Adult suffering from Coronavirus Disease 2019 Pneumonia according to the diagnostic criteria in effect at the inclusion time or very strongly suspected 2. Patient treated by High Flow Nasal Therapy 3. Moderate or severe Acute Respiratory Distress Syndrome: Bilateral Radiological Opacities not entirely explained by Atelectasis, Nodules, or Effusions; Acute hypoxaemia with worsening within the previous week, not explained entirely by left ventricular failure; Arterial Partial Pressure of Oxygen to Fraction of Inspired Oxygen ratio less three hundred millimeters of mercury (or equivalent Peripheral Oxygen Saturation to Fraction of Inspired Oxygen ratio) 4. Oral consent | 1. High Flow Nasal Cannula adapted for a Peripheral Oxygen Saturation of ninety to ninety-five percent 2. Initial Minimum Flow Rate of fifty liters per minute unless poorly tolerated 3. High Flow Nasal Cannula 1^st^ weaned by decreasing the Fraction of Inspired Oxygen to 0.4, followed by Flow Rate Reduction 4. Attempt made to switch to standard oxygen therapy at four to six liters per minute in clinically stable patients with a Fraction of Inspired Oxygen less than or equal to 0.4 and a flow rate less than or equal to thirty liters per minute | 1. Patients underwent Awake-Prone Positioning for the maximum amount of time tolerable 2. Awake-Prone Positioning was stopped upon High Flow Nasal Cannula weaning, discharge, Endotracheal Intubation, or death 3. Endotracheal Intubation initiated upon worsening respiratory failure (Respiratory Rate greater than forty breaths per minute, respiratory muscle weakness, respiratory acidosis with a pH of 7.25, excessive tracheal secretions, Peripheral Oxygen Saturation less than ninety percent even if the Fraction of Inspired Oxygen is greater than or equal to 0.8, hemodynamically unstable, or worsening mentation) | 1. High Flow Nasal Cannula with the intent of achieving a Peripheral Oxygen Saturation of ninety to ninety-five percent 2. Usual Care | Twenty-eight days |
| Javed (Pakistan) | 1. Confirmed Coronavirus Disease 2019 Pneumonia/Acute Respiratory Distress Syndrome cases | 1. Simple Mask 2. Non-Rebreather Mask 3. Non-Invasive Ventilation | 1. Intermittent Awake-Prone Positioning | 1. Institutional Protocol | Ninety days |
| Ehrmann (United States) | 1. Coronavirus Disease 2019 Pneumonia based on CDC guidelines 2. Acute Hypoxaemic Respiratory Failure 3. Acute onset within a week of insult, or new (within a week) or worsening respiratory symptoms 4. Bilateral opacities on Chest X-Ray and Computed Tomography Scan not fully explained by effusions, lobar or lung collapse, or nodules 5. Cardiac Failure, not the Primary Acute Respiratory Failure cause 6. Written informed consent 7. Arterial Partial Pressure of Oxygen to Fraction of Inspired Oxygen ratio less than two hundred millimeters of mercury or Peripheral Oxygen Saturation to Fraction of Inspired Oxygen ratio less than two hundred forty at fifty liters per minute and a Peripheral Oxygen Saturation maintained at ninety-two to ninety-five percent | 1. High Flow Nasal Cannula initiated at fifty liters per minute and thirty-seven degrees Celsius 2. Nasal Cannula size determined by patient’s nostril size 3. Fraction of Inspired Oxygen adjusted to maintain a Peripheral Oxygen Saturation of ninety-two to ninety-five percent 4. Flow and Temperature adjusted based on the patient’s Comfort and Clinical Response | 1. Patients underwent Awake-Prone Positioning for the maximum amount of time tolerable 2. Awake-Prone Positioning was stopped upon High Flow Nasal Cannula weaning, discharge, Endotracheal Intubation, or death 3. Endotracheal Intubation initiated upon worsening Respiratory Failure (Respiratory Rate less than forty breaths per minute, respiratory muscle weakness, respiratory acidosis with a pH less than 7.25, excessive tracheal secretions, Peripheral Oxygen Saturation less than ninety percent even if the Fraction of Inspired Oxygen is greater than or equal to 0.8, hemodynamically unstable, or worsening mentation) | 1. High Flow Nasal Cannula with the intent of achieving a Peripheral Oxygen Saturation of ninety to ninety-five percent 2. Usual Care | Twenty-eight days |
| Johnson (United States) | 1. At least eighteen years old 2. Suspected or Confirmed Coronavirus Disease 2019 3. Scheduled for admission or already admitted to an inpatient hospital bed 4. Patients had to have been enrolled within two days of hospital admission | 1. Room Air 2. Nasal Cannula 3. High Flow Nasal Cannula | 1. Awake-Prone Positioning during the daytime 2. Also given the option to be in the left or right lateral positions 3. Massage therapy cushion offered for comfort 4. Any preferred position during the nighttime | 1. Usual Care | Three days |
| Taylor (United States) | 1. Admitted by one of the study teams 2. Tested positive for Severe Acute Respiratory Syndrome Coronavirus 2 within one week or suspected to have Coronavirus Disease 2019 pneumonia 3. Experienced a Peripheral Oxygen Saturation less than ninety-three percent on room air an oxygen requirement of at least three liters per minute without the need for mechanical ventilation | 1. Room Air 2. Less than four liters per minute on Nasal Cannula 3. Four to six liters per minute on Nasal Cannula 4. Medium Flow Nasal Cannula 5. Humidified High Flow Nasal Cannula 6. Bilevel Positive Pressure Ventilation | 1. Patients and clinicians taking part in the intervention underwent phone interviews two days before Awake-Prone Positioning commencement 2. Patients and clinicians were given educational material regarding Awake-Prone Positioning 3. Patients were encouraged to undergo Awake-Prone Positioning for as long as possible 4. Awake-Prone Positioning ceased after at least two days, Endotracheal Intubation, Intensive Care Unit transfer, or death | 1. Usual Care | Not Applicable |
| Fralick (Canada and the United States) | 1. At least eighteen years old 2. Coronavirus Disease 2019 suspected by treating clinician or confirmed by diagnostic test 3. Able to lie Prone with verbal instruction 4. Requiring Supplemental Oxygen less than or equal to fifty percent Fraction of Inspired Oxygen 5. Capable of making treatment-related decisions 6. Hospitalized in the last two days with suspected or confirmed Coronavirus Disease 2019 infection or diagnosed with a Nosocomial infection in the last two days during hospital stay | 1. Nasal Prongs | 1. Patients were instructed to undergo Awake-Prone Positioning for a week or until the first patient was discharged or no longer required supplemental oxygen for more than one day | 1. Usual Care (Supine) | Thirty days |
| Ehrmann (Canada) | 1. Coronavirus Disease 2019 Pneumonia based on Centers for Disease Control guidelines 2. Acute Hypoxaemic Respiratory Failure 3. Acute onset within 1 week of insult, or new (within 1 week) or worsening Respiratory Symptoms 4. Bilateral Opacities on Chest X-Ray and Computed Tomography Scan not fully explained by Effusions, Lobar or lung collapse, or Nodules 5. Cardiac Failure, not the primary Acute Respiratory Failure Cause 6. Written Informed Consent 7. Arterial Partial Pressure of Oxygen to Fraction of Inspired Oxygen ratio less than two hundred millimeters of mercury or Peripheral Oxygen Saturation to Fraction of Inspired OXygen ratio less than two hundred forty at fifty liters per minute, and Peripheral Oxygen Saturation maintained at ninety-two to ninety-five percent | 1. High Flow Nasal Cannula initiated at fifty liters per minute and thirty-seven degrees Celsius 2. Nasal Cannula size as determined by the patient’s nostril size 3. Fraction of Inspired Oxygen adjusted to maintain a Peripheral Oxygen Saturation of ninety-two to ninety-five percent 4. Flow and temperature adjusted based on the patient’s comfort and clinical response | 1. Patients underwent Awake-Prone Positioning for the maximum amount of time tolerable 2. Awake-Prone Positioning was stopped upon High Flow Nasal Cannula weaning, discharge, Endotracheal Intubation, or death 3. Endotracheal Intubation initiated upon worsening respiratory failure (Respiratory Rate less than forty breaths per minute., respiratory muscle weakness, respiratory acidosis with a pH less than 7.25, excessive tracheal secretions, Peripheral Oxygen Saturation less than ninety percent even if the Fraction of Inspired Oxygen is greater than or equal to 0.8, hemodynamically unstable, or worsening mentation) | 1. High Flow Nasal Cannula with the intent of achieving a Peripheral Oxygen Saturation of ninety to ninety-five percent 2. Usual Care | Twenty-eight days |
| Kharat (Switzerland) | 1. At least eighteen years old 2. Admitted to ward for treatment of Coronavirus Disease 2019 Pneumonia with low-flow oxygen therapy (one to six liters per minute) through Nasal Cannula to achieve a Peripheral Oxygen Saturation of ninety to ninety-two percent | 1. Low-Flow Oxygen Therapy (one to six liters per minute) on Nasal Cannula | 1. Patients were given a brochure with instructions regarding Awake-Prone Positioning 2. Patients timed themselves with their smartphones and changed position every four hours 3. Vital signs were recorded daily 4. Patients were surveyed daily regarding Awake-Prone Positioning duration and tolerance | 1. Oxygen titration on Nasal Cannula to achieve a Peripheral Oxygen Saturation of ninety to ninety-four percent and monitored six times per day 2. Empirical antibiotics for community-acquired pneumonia 3. Fluid restriction 4. Hydroxychloroquine and lopinavir/ritonavir as needed | One day |
| Rosén (Sweden) | 1. At least eighteen years old 2. Coronavirus Disease 2019 verified by positive Severe Acute Respiratory Syndrome-Coronavirus 2 Reverse Transcriptase-Polymerase Chain Reaction test on nasopharyngeal or oropharyngeal swab 3. Hypoxaemic respiratory failure 4. High Flow Nasal Oxygen or Non-Invasive Ventilation 5. Arterial Partial Pressure of Oxygen to Fraction of Inspired Oxygen ratio less than or equal to twenty kilopascals (one hundred fifty millimeters of mercury) or corresponding Peripheral Oxygen Saturation and Fraction of Inspired Oxygen for more than one hour | 1. High-Flow Nasal Oxygen 2. Non-Invasive Ventilation | 1. Patients were encouraged to undergo Awake-Prone Positioning and be in a semi-prone or lateral position during breaks 2. Awake-Prone Positioning was stopped if patients needed to be intubated, died, or received oxygen at a rate of five liters per minute for twelve hours on Nasal Cannula or open face mask 3. Awake-Prone Positioning could also be stopped anytime at the attending’s discretion | 1. Patients were assigned to undergo usual care 2. Awake-Prone Positioning only if approved by the attending | Thirty days |
| Ehrmann (Ireland) | 1. Suspected or confirmed Coronavirus Disease 2019 infection 2. Bilateral Infiltrates on Chest X-Ray 3. Peripheral Oxygen Saturation less than ninety-four percent on a forty percent Fraction of Inspired Oxygen by either a Venturi Facemask or High Flow Nasal Cannula 4. Respiratory Rate less than forty breaths per minute 5. Written informed consent | 1. Standard care | 1. Patients underwent Awake-Prone Positioning for the maximum amount of time tolerable 2. Awake-Prone Positioning was stopped upon High Flow Nasal Cannula weaning, discharge, Endotracheal Intubation, or death 3. Endotracheal Intubation initiated upon worsening respiratory failure (Respiratory Rate less than forty per minute, respiratory muscle weakness, respiratory acidosis with a pH less than 7.25, excessive tracheal secretions, Peripheral Oxygen Saturation less ninety percent even if the Fraction of Inspired Oxygen is greater than or equal to 0.8, hemodynamically unstable, or worsening mentation) | 1. High Flow Nasal Cannula with the intent of achieving a Peripheral Oxygen Saturation of ninety to ninety-five percent 2. Usual Care | Twenty-eight days |

Supplemental Table 2 - Secondary Outcome(s) and Conclusions of included RCTs

| **1^st^ Author (Nation(s))** | **Secondary Outcome(s)** | **Conclusion(s)** |
| --- | --- | --- |
| Harris (Qatar) | 1. Changes in Physiology (Respiratory Rate, Peripheral Oxygen Saturation, Fraction of Inspired Oxygen, Pulse, and Blood Pressure) 2. Duration of Awake-Prone Positioning 3. Thirty-day mortality 4. Intensive Care Unit Length of Stay 5. Hospital Length of Stay 6. Use of Rescue PRone 7. Prone Failures 8. Harm associated with Awake-Prone Positioning (Skin Breakdown, Pressure Areas, Displaced Medical Devices, Muscle/Back Pain requiring Analgesia, Nausea/Vomiting, and Haemodynamic Instability) | 1. Awake-Prone Positioning did not reduce the need to escalate Oxygen support 2. Awake-Prone Positioning did not improve respiratory physiology |
| Alhazzani (Canada, Kuwait, South Africa, and the United States) | 1. Mortality at sixty days 2. Free from Invasive Mechanical Ventilation at thirty days 3. Non-Invasive Ventilation free days 4. Hospital Length of Stay truncated at sixty days 5. Days alive and outside of the hospital truncated at sixty days 6. Proning complications (Accidental IV removal, hypotension, falls, pressure ulcer, or other adverse event) | 1. Awake-Prone Positioning was not associated with a significant Endotracheal Intubation rate reduction after thirty days 2. Effect size is imprecise due to the small sample size |
| Ehrmann (Mexico) | 1. Days spent in the hospital 2. Mortality 3. Endotracheal rate 4. Invasive ventilation duration for intubated patients who survive at four weeks 5. Intubated patient mortality 6. High Flow Nasal Cannula therapy use length among those for whom High Flow Nasal Cannula was successful 7. Time to Non-Invasive Ventilation, Endotracheal Intubation, or death 8. Awake-Prone Positioning response 9. Daily Awake-Prone Positioning duration within the first two weeks after enrollment 10. Crossover number | 1. Awake-Prone Positioning was associated with statistically significant Endotracheal Intubation and death rate reductions after twenty-eight days |
| Liu (China) | 1. Awake-Prone Positioning duration (hours per day) 2. Mortality 3. Intermediate Care Unit and Hospital Length of Stay 4. Need for Respiratory Support, Continuous Renal Replacement Therapy, and/or Vasopressors | 1. Prolonged Awake Prone Positioning had a favourable effect on intubation and mortality within twenty eight days of enrollment when compared with shorter durations |
| Nasrallah (Egypt) | 1. Comparative Frequency of complications from Awake-Prone Positioning 2. Intensive Care Unit Length of Stay 3. Mortality Rate during the first four weeks of Surgical Intensive Care Unit Admission | 1. Oxygen through a High Flow Nasal Cannula coupled with Awake-Prone Positioning decreased the Endotracheal Intubation rate in patients with mild Acute Respiratory Distress Syndrome in the Intensive Care Unit when compared to patients managed only with High Flow Nasal Cannula 2. Oxygen through a High Flow Nasal Cannula coupled with Awake-Prone Positioning decreased the Intensive Care Unit Length of Stay in patients with mild Acute Respiratory Distress Syndrome when compared to those managed only with High Flow Nasal Cannula 3. Oxygen through a High Flow Nasal Cannula coupled with Awake-PRone Positioning improved the outcome of patients with mild Acute Respiratory Distress Syndrome when compared to those managed with High Flow NAsal Cannula only |
| Gad (Egypt) | 1. Reduction in Intensive Care Unit Hospital Length of Stay | 1. Prone positioning and Non-Invasive Ventilation showed a marked improvement in the Arterial Partial of Oxygen and Peripheral Oxygen Saturation in Coronavirus Disease 2019 patients, with superiority of Non-Invasive Ventilation in those who were hypercapnic or those unable to carry out work of breathing with improvement in clinical symptoms 2. In comparing both groups, rate of conversation of severe Coronavirus Disease 2019 to critically ill and avoidance of invasive ventilation were not statistically significant. |
| Gopalakrishnan (India) | 1. Mechanical Ventilation 2. Time to Endotracheal Intubation 3. Progression to High Flow Nasal Oxygen/Non-Invasive Ventilation 4. Duration of Hospital Stay 5. Mortality 6. Change in Peripheral Oxygen Saturation to Fraction of Inspired Oxygen ratio 7. Change in Arterial Partial Pressure of Oxygen to Fraction of Inspired Oxygen ratio | 1. Early Awake-Prone Positioning did not decrease thirty-day mortality in patients with moderate Coronavirus Disease 2019 hypoxaemia 2. Early Awake-Prone Positioning did not decrease the need for Endotracheal Intubation in patients with moderate Coronavirus Disease 2019 hypoxaemia 3. Early Awake-Prone Positioning did not decrease the time to Endotracheal Intubation in patients with moderate Coronavirus Disease 2019 hypoxaemia |
| Jayakumar (India) | 1. Respiratory Support Escalation Proportion 2. Awake-Prone Positioning duration (hours) 3. Maximum continuous Awake-Prone Positioning duration (hours) in a given day 4. Intensive Care Unit Length of Stay 5. Intensive Care Unit Mortality 6. Adverse Events 7. Reasons for not undergoing Awake-Prone Positioning | 1. Awake-Prone Positioning is safe despite low adherence 2. Future Randomised-Control Trials should be completed in such a way that patients are recruited early, crossover is minimized, and Awake-Prone Positioning duration is increased |
| Ehrmann (France) | 1. Days spent in the hospital 2. Mortality 3. Endotracheal Intubation rate 4. Invasive ventilation duration for intubated patients who survive at four weeks 5. Intubated patient mortality 6. High Flow Nasal Cannula therapy use length among those for whom High Flow Nasal Cannula was successful 7. Time to Non-Invasive Ventilation, Endotracheal Intubation, or death 8. Awale-Prone Positioning response 9. Daily Awake-Prone Positioning duration within the first two weeks after enrollment 10. Crossover number | 1. Awake-Prone Positioning was associated with statistically significant Endotracheal Intubation and death rate reductions after twenty-eight days |
| Yarahmadi (Iran) | 1. Hospital stays, intubation rate, and survival | 1. Prone Positioning can be considered in awake non intubated patients with Coronavirus Disease 2019 to improve short-term clinical outcomes such as respiratory and haemodynamic status |
| Hashemian (Iran) | 1. Intensive Care Unit Length of Stay 2. Need for Endotracheal Intubation | 1. Awake-Prone Positioning and Non-Invasive Ventilation are favored over Non-Invasive Ventilation alone |
| Rampon (Spain and the United States) | 1. Mechanical Ventilation 2. Hospital Mortality 3. Acute Respiratory Distress Syndrome Diagnosis 4. Median self-reported dyspnea level 5. Self-reported Awake-Prone Positioning discomfort degree 6. Venous catheter loss 7. Urinary catheter loss | 1. Receipt of text messages with Awake-Prone Positioning instructions was not associated with an increased Awake-Prone Positioning adherence |
| Ehrmann (Spain) | 1. Days spent in the hospital 2. Mortality 3. Endotracheal Intubation rate 4. Invasive ventilation duration for intubated patients who survive at four weeks 5. Intubated patient mortality 6. High Flow Nasal Cannula therapy use length among those for whom High Flow Nasal Cannula was successful 7. Time to Non-Invasive Ventilation, Endotracheal Intubation, or death 8. Awake-Prone Positioning response 9. Daily Awake-Prone Positioning duration within the first two weeks after enrollment 10. Crossover number | 1. Awake-Prone Positioning was associated with statistically significant Endotracheal Intubation and death rate reductions after twenty-eight days |
| Javed (Pakistan) | 1. Pneumonia Severity Index (PSI) 2. Arterial Partial Pressure of Oxygen 3. Supplemental Oxygen Therapy Mask Type | 1. Awake-Prone Positioning for one week did not yield a statistically significant mortality benefit 2. A mortality benefit may be found with increased sample size and/or total Awake-Prone Positioning duration |
| Ehrmann (United States) | 1. Days spent in the hospital 2. Mortality 3. Endotracheal Intubation rate 4. Invasive ventilation duration for intubated patients who survive at four weeks 5. Intubated patient mortality 6. High Flow Nasal Cannula therapy use length among those for whom High Flow Nasal Cannula was successful 7. Time to Non-Invasive Ventilation, Endotracheal Intubation, or death 8. Awake-Prone Positioning response 9. Daily Awake-Prone Positioning duration within the first two weeks after enrollment 10. Crossover number | 1. Awake-Prone Positioning was associated with statistically significant Endotracheal Intubation and death rate reductions after twenty-eight days |
| Johnson (United States) | 1. Change in the Arterial Partial Pressure of Oxygen to Fraction of Inspired Oxygen ratio after two days 2. Length of Stay 3. Required Oxygen Delivery System Escalation 4. Maximal Amount of Oxygen Support on Room Air, Nasal Cannula, High Flow Nasal Cannula, and Endotracheal Intubation 5. Number of patients transferred to the Intensive Care Unit 6. Number of patients requiring Endotracheal Intubation 7. In-Hospital Mortality 8. Number of Ventilator-Free Days | 1. Awake-Prone Positioning is not feasible 2. No oxygenation improvements were observed after two and three days |
| Taylor (United States) | 1. Number of patients who attempted Awake-Prone Positioning within two days 2. Number of patients who required oxygen support two days after baseline 3. Number of patients who required Adjunctive therapies 4. Number of patients who experienced adverse events within two days 5. Number of patients admitted to the Intensive Care Unit within two days 6. Number of patients admitted to Intensive Care Unit during hospitalization 7. Median Hospital Length of Stay 8. Discharge Disposition | 1. A Randomised-Control Trial on the effects of Awake-Prone Positioning is necessary |
| Fralick (Canada and the United States) | 1. Hospitalisation Length 2. Awake-Prone Positioning Adverse Events 3. Change in the Peripheral Oxygen Saturation to Fraction of Inspired Oxygen ratio | 1. Awake-Prone Positioning was not associated with statistically significant Endotracheal Intubation, death, or respiratory failure rate reductions. 2. Neither a benefit nor harm could be found because the confidence intervals included the null value. 3. Awake-Prone Positioning adherence was quite poor, and newer methods are needed to increase adherence |
| Ehrmann (Canada) | 1. Days spent in the hospital 2. Mortality 3. Endotracheal Intubation rate 4. Invasive ventilation duration for intubated patients who survive at four weeks 5. Intubated patient mortality 6. High Flow Nasal Cannula therapy use length among those for whom it was successful 7. Time to Non-Invasive Ventilation, Endotracheal Intubation, or death 8. Awake-Prone Positioning response 9. Daily Awake-Prone Positioning duration within the first two weeks after enrollment 10. Crossover number | 1. Awake-Prone Positioning was associated with statistically significant Endotracheal Intubation and death rate reductions after twenty-eight days |
| Kharat (Switzerland) | 1. Peripheral Oxygen Saturation to Fraction of Inspired Oxygen ratio after one day 2. Respiratory Rate after one day 3. Heart Rate after one day 4. Patient Trajectory 5. Potential Adverse Effects of Awake-Prone Positioning | 1. Awake-Prone Positioning was associated with a decreased oxygen requirement; however, the effect was not statistically significant due to the small sample size 2. The decreased oxygen requirement after one day was encouraging 3. Another Randomised-Control Trial with a larger sample size is needed |
| Rosén (Sweden) | 1. Daily Awake-Prone Positioning duration 2. Total Protocol Duration 3. Daily Awake-Prone Positioning Duration (during the first three days) 4. Thirty-day Mortality 5. Ventilator Free Days in all patients 6. Ventilator-Free Days in Endotracheally Intubated Patients 7. High-Flow Nasal Oxygen/Non-Invasive Ventilation Free Days 8. Time from Enrollment to Invasive Mechanical Ventilation (IMV) 9. Number of patients admitted to the Intensive Care Unit 10. Intensive Care Unit Length of Stay 11. Intensive Care Unit-Free Days 12. Hospital Length of Stay 13. Number of patients who received vasoactive drugs 14. Number of patients sedated by continuous infusion 15. Number of patients who received Renal Replacement Therapy 16. Number of patients who received Extracorporeal Membrane Oxygenation (ECMO) 17. World Health Organization (WHO) Clinical Progression Scale after one week 18. WHO Clinical Progression Scale after thirty days 19. Number of patients who experienced adverse events | 1. The intervention protocol yielded an increased Awake-Prone Positioning duration |
| Ehrmann (Ireland) | 1. Days spent in the hospital 2. Mortality 3. Endotracheal Intubation rate 4. Invasive ventilation duration for intubated patients who survive at four weeks 5. Intubated patient mortality 6. High Flow Nasal Cannula therapy use length among those for whom High Flow Nasal Cannula was successful 7. Time to Non-Invasive Ventilation, Endotracheal Intubation, or death 8. Awake-Prone Positioning response 9. Daily Awake-Prone Positioning duration within the first two weeks after enrollment 10. Crossover number | 1. Awake-Prone Positioning was associated with statistically significant Endotracheal Intubation and death rate reductions after twenty-eight days |

Supplemental Figure 1 – Risk of Bias


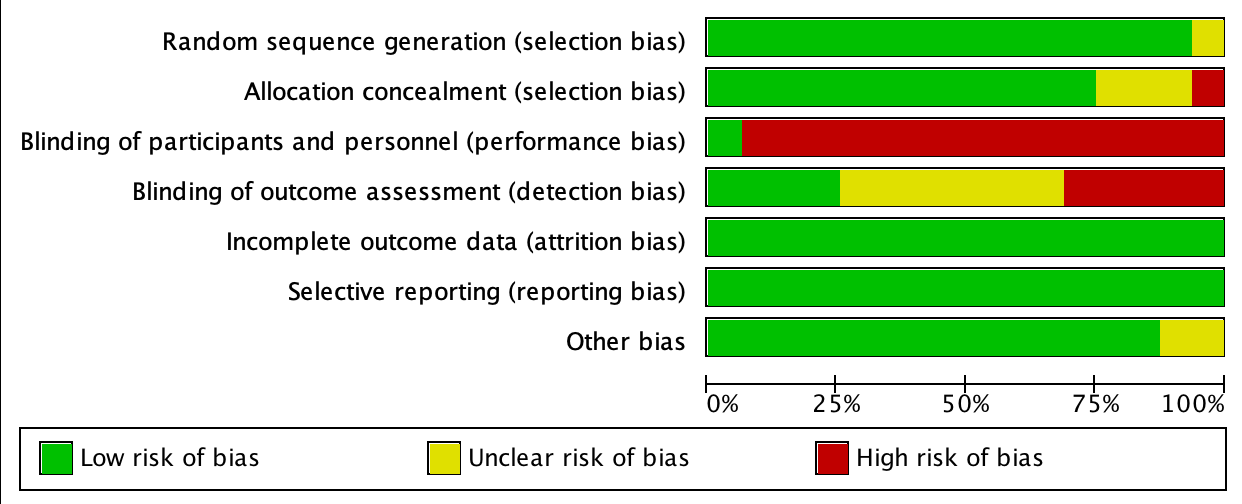


Supplemental Figure 2 – Risk of Bias Summary


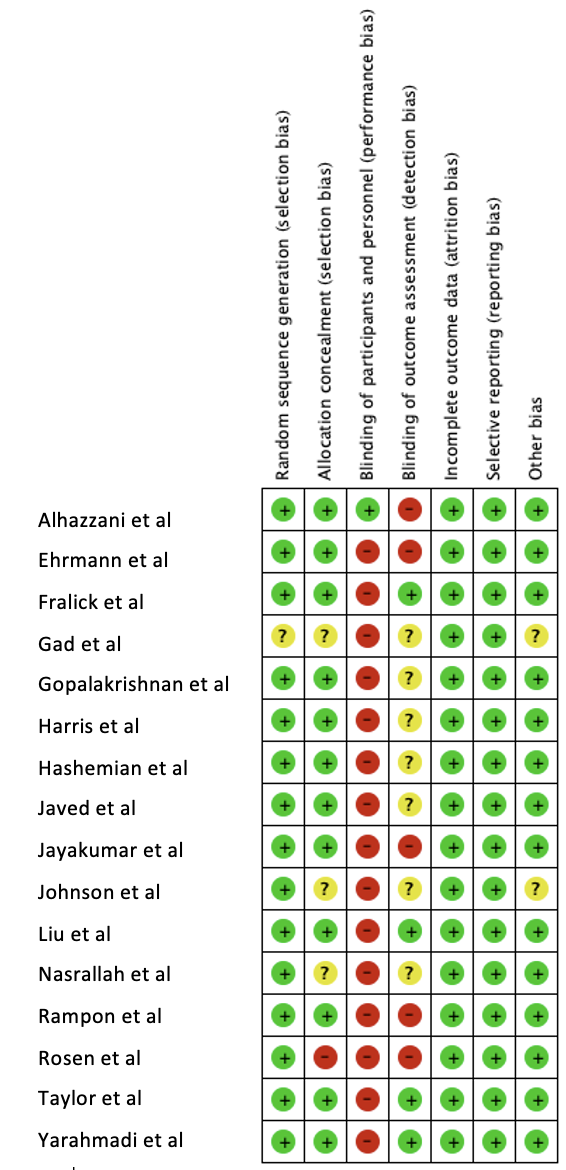


Supplemental Figure 3 - Sensitivity Analysis for the Outcome of Endotracheal Intubation Excluding Study with High Risks of Bias


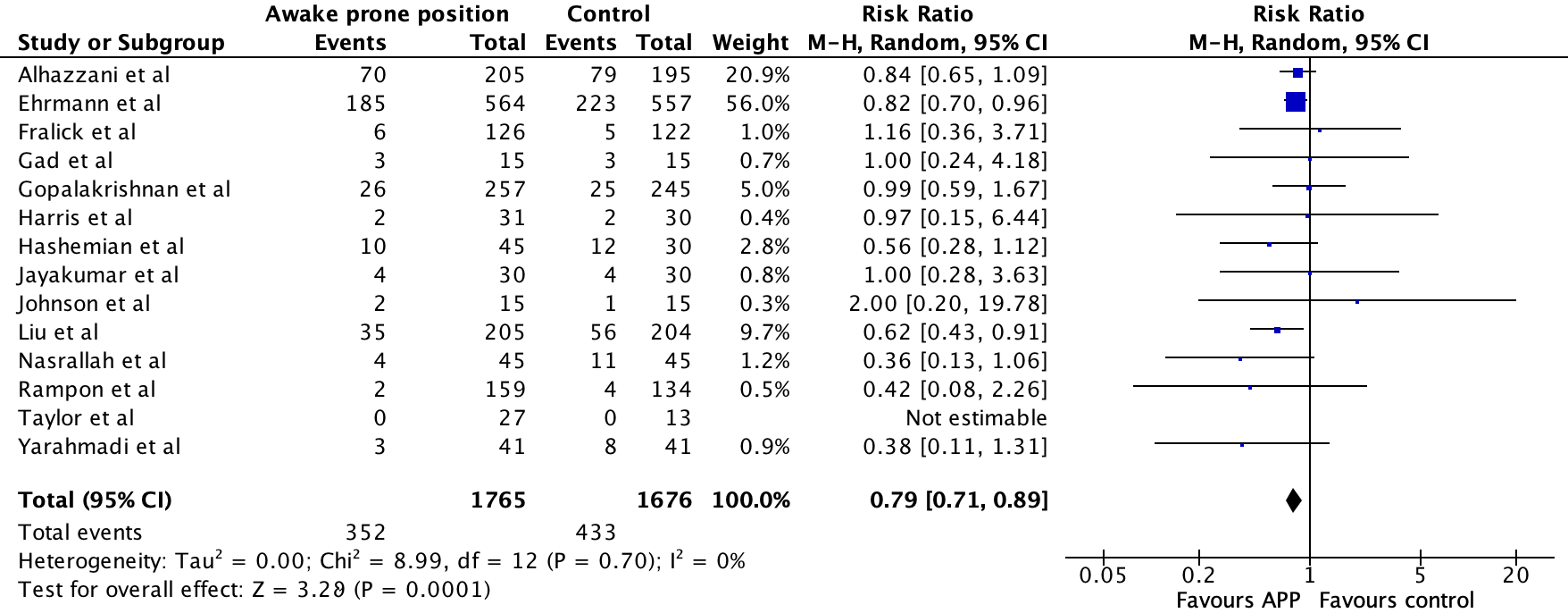


Supplemental Figure 4 - Sensitivity analysis for the Outcome of Endotracheal Intubation excluding Studies with Unclear and High Risks of Bias


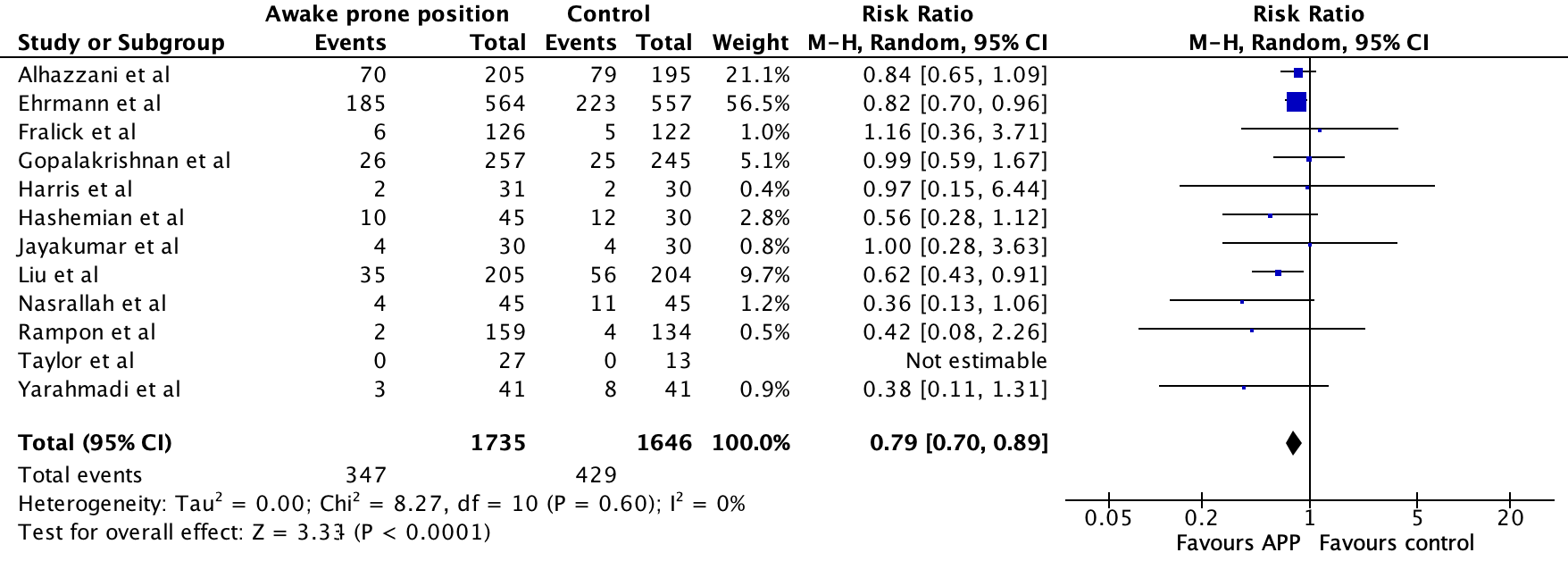


Supplement Figure 5 - Forest plot for risk of intubation stratified by duration of APP (median or mean duration <8h/day vs >/=8h/day)


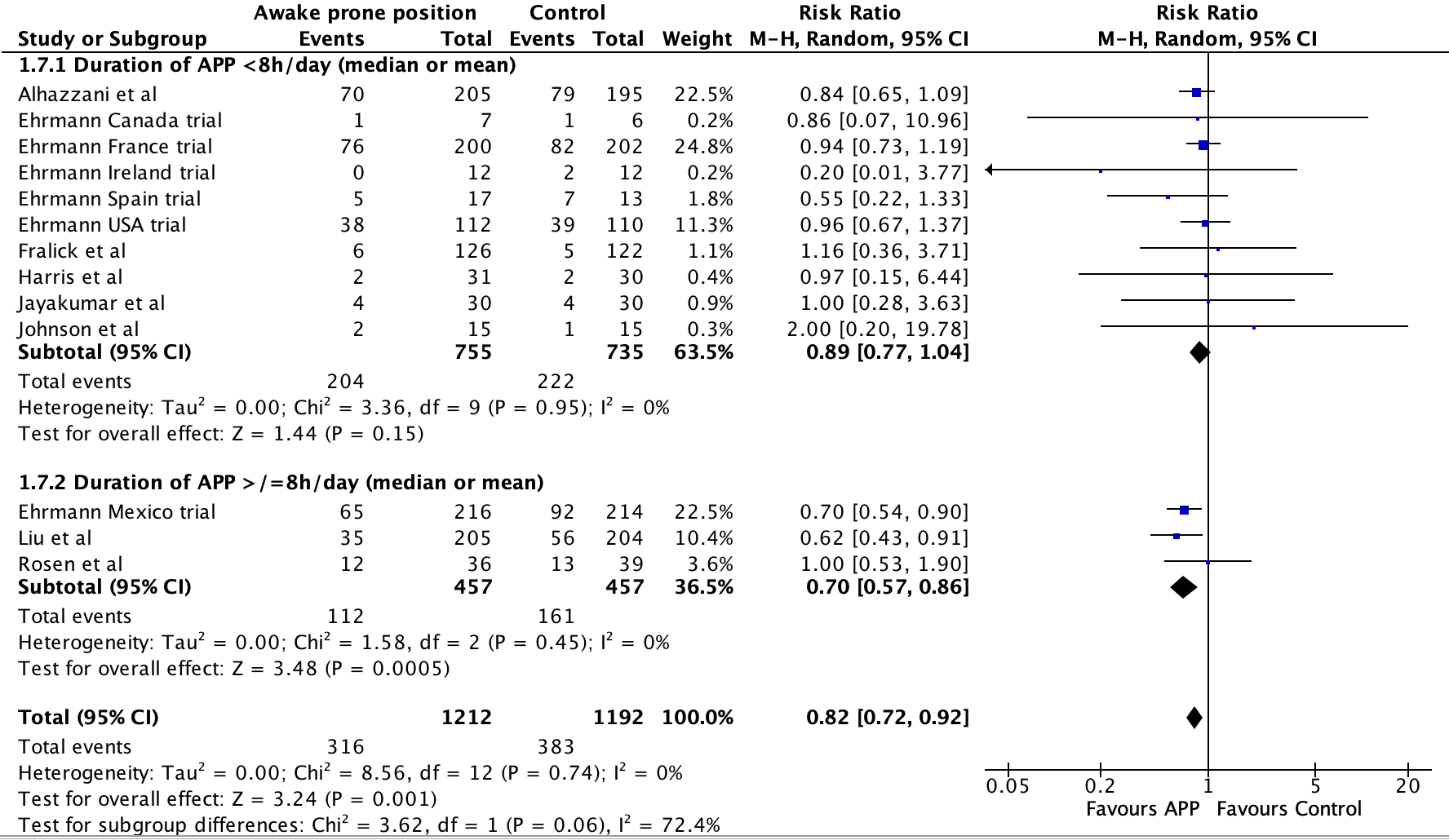


Supplemental Figure 6 - Forest Plot analysis comparing mortality in countries stratified by Power Distance Index


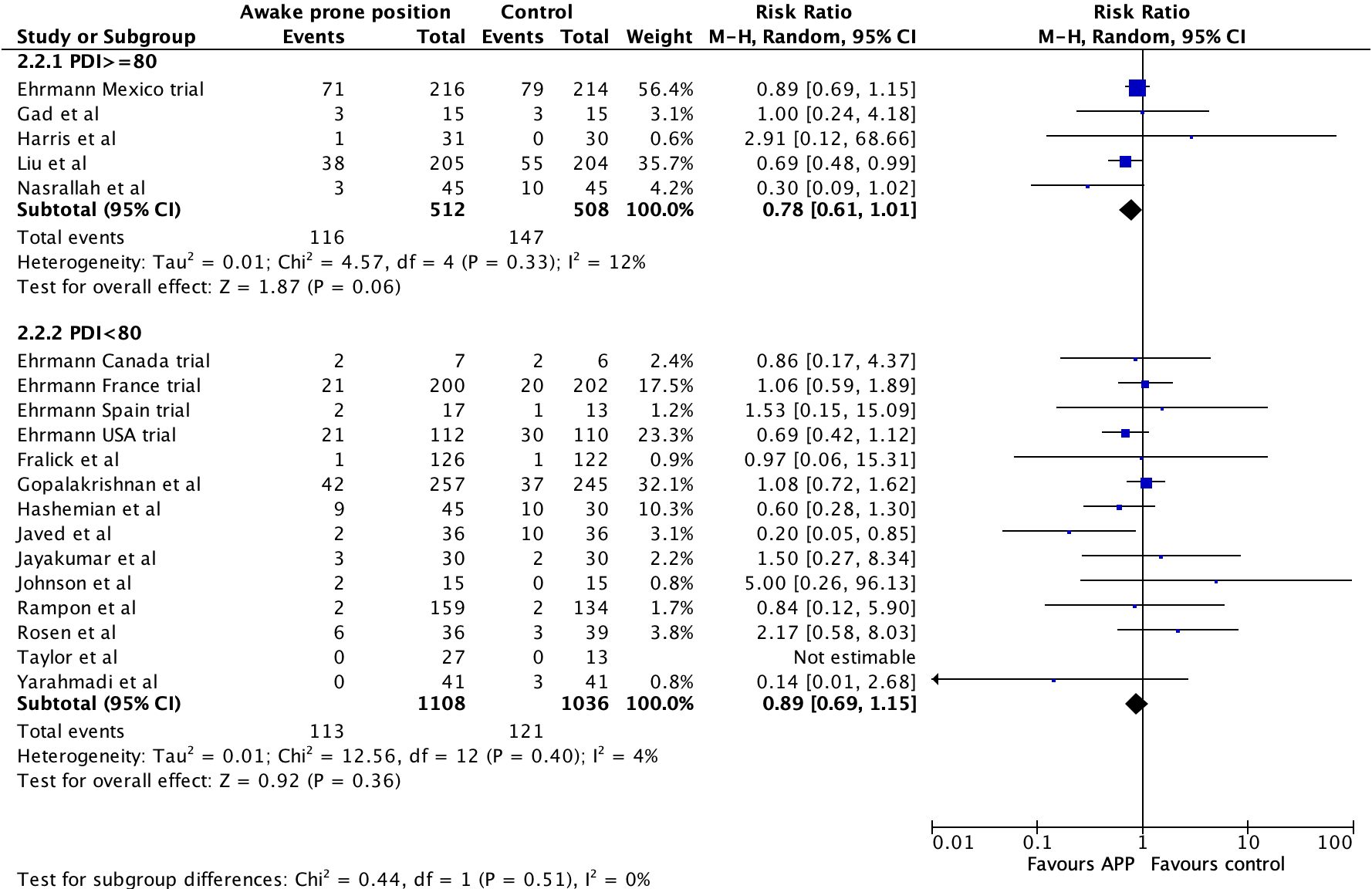


Supplemental Figure 7 - Graph representing relationship between PDI and Daily duration of APP

Supplement Table 3 - Relation between duration of APP and PDI

|  | Median duration of APP >/=8h | Median duration of APP < 8h |
| --- | --- | --- |
| PDI>/=80 | 2 | 1 |
| PDI<80 | 1 | 9 |

The chi-square statistic is 4.1744. The *p*-value is .041038. Significant at *p* < .05.

Supplement Figure 8 - Forest Plot comparing the Escalation of Respiratory Support in the Awake-Prone Positioning and Usual Care groups


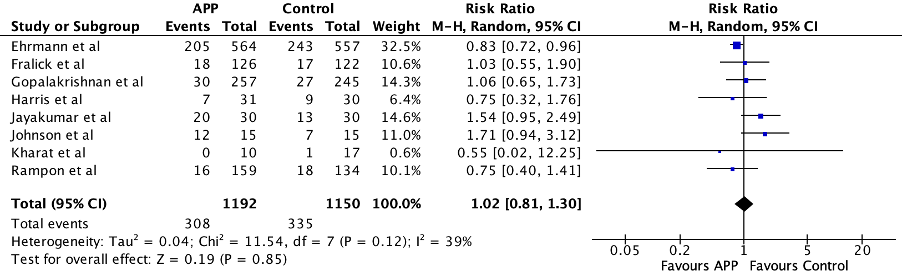


Supplemental Figure 9 - Forest Plot comparing the Need for Intensive Care Unit Admission in the Awake-Prone Positioning and Usual Care groups


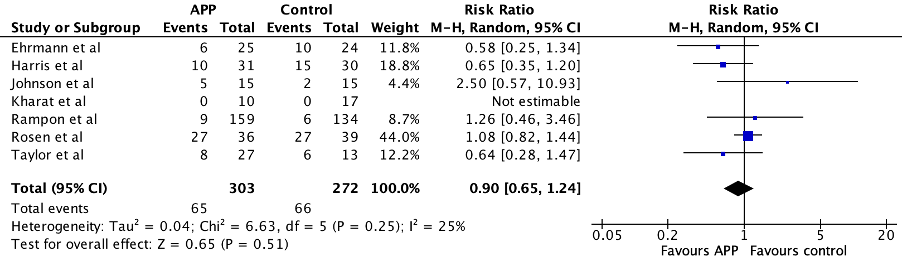


Supplemental Figure 10 - Sensitivity Analysis for Mortality excluding Study with High Risks of Bias


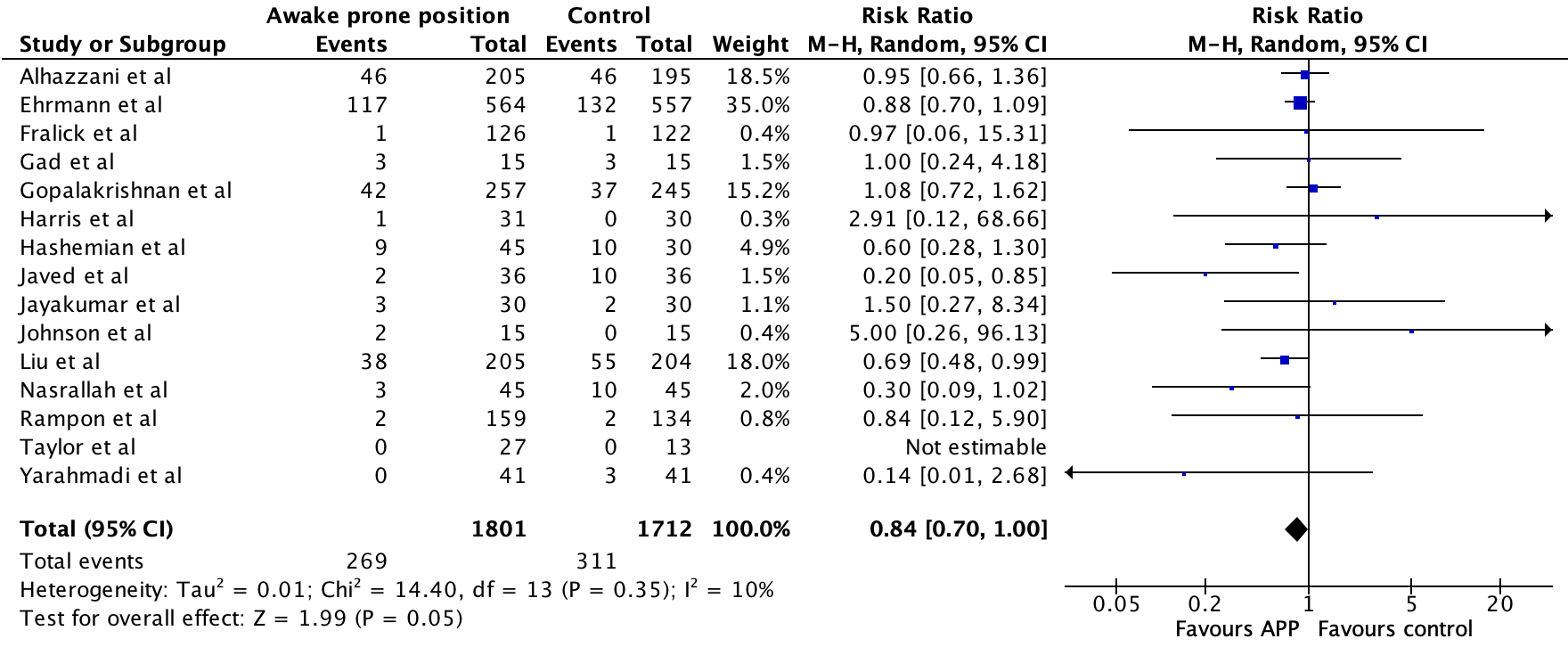


Supplemental Figure 11 - Sensitivity Analysis for Mortality excluding Studies with Unclear and High Risks of Bias


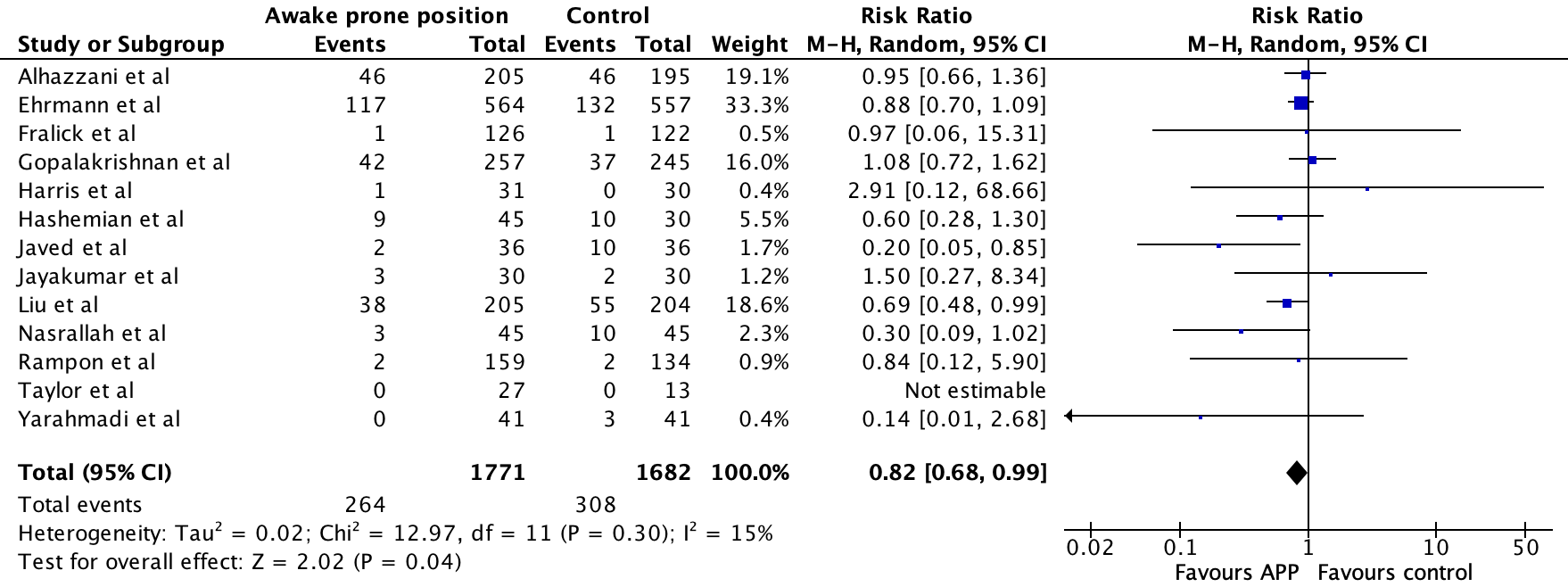


Supplemental Figure 12 - Funnel Plot of studies comparing Endotracheal Intubation in the Awake-Prone Positioning and Usual Care groups

**
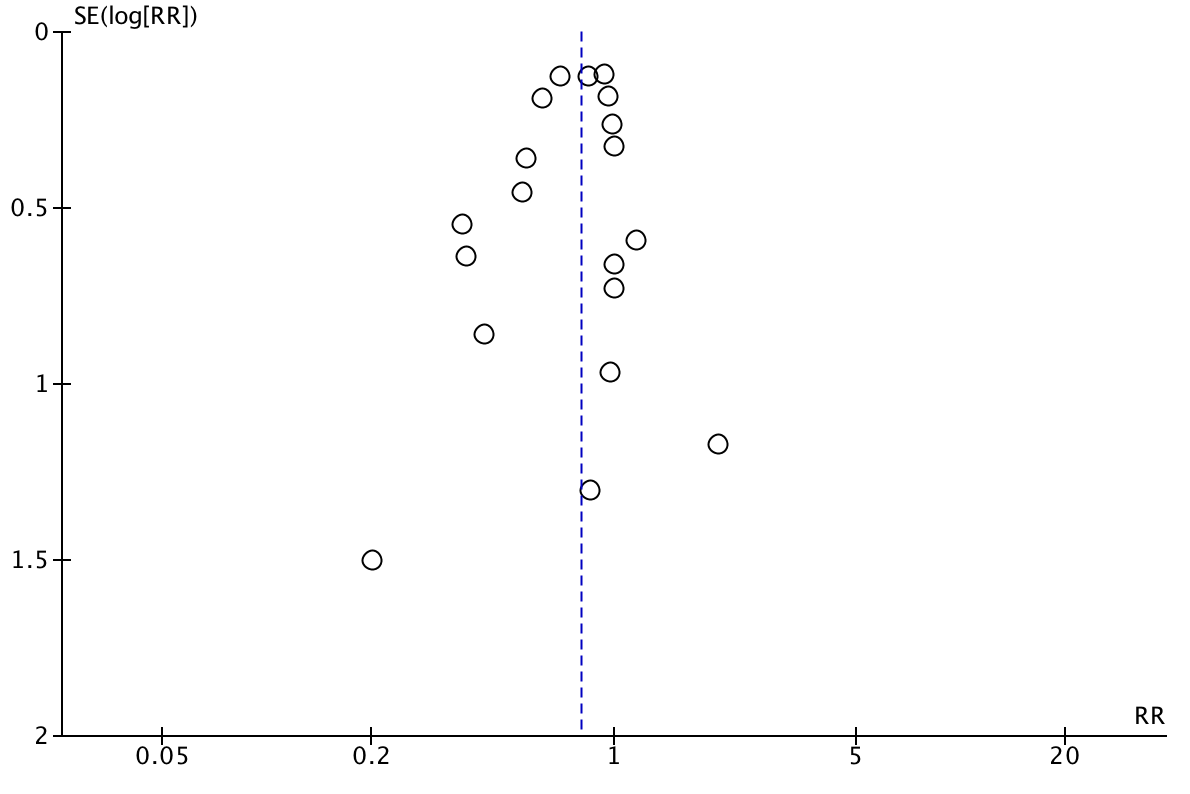
**

Supplemental Figure 13 - Funnel Plot of studies comparing Mortality in the Awake-Prone Positioning and Usual Care groups


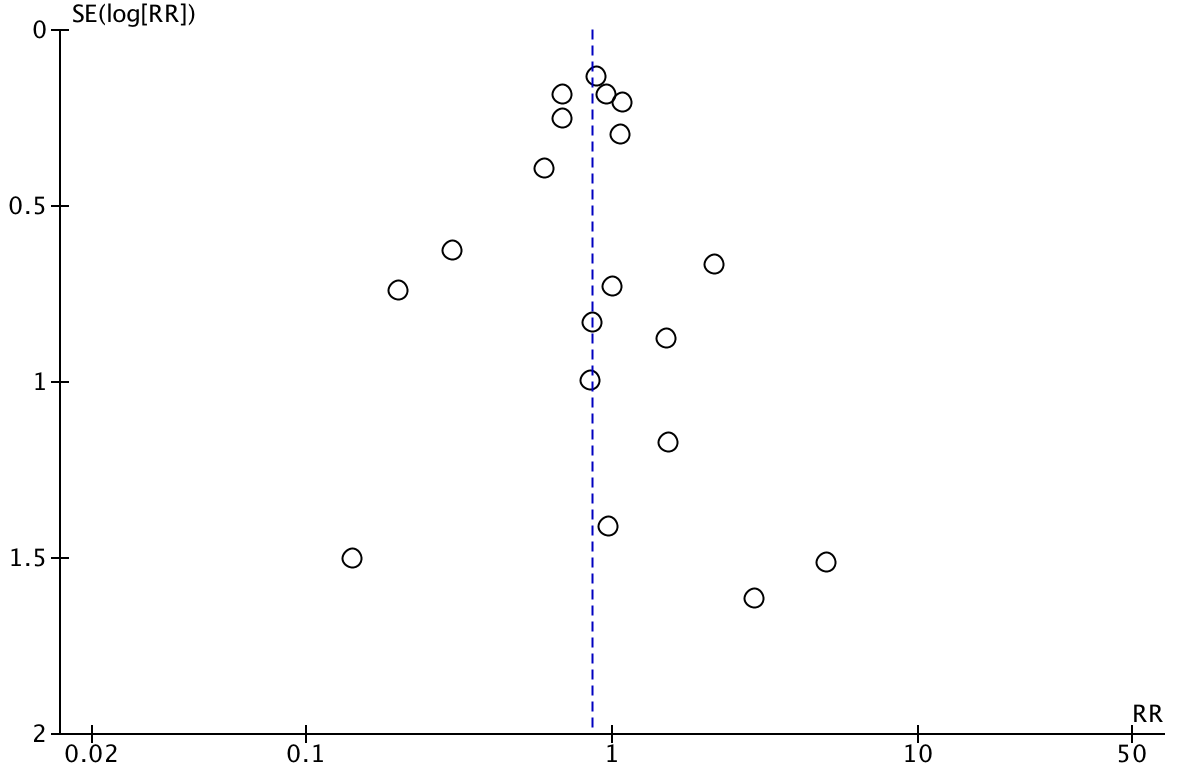


Supplemental Table 4 – Summary of Adverse events mentioned in the included RCTs

|  | Adverse events | |
| --- | --- | --- |
| **Alhazzani et al** | APP (205) | Control (205) |
| Pain or discomfort | 16 | 0 |
| Desaturation | 4 | 0 |
| Unintentional removal of intravenous access | 1 | 0 |
| Hypotension | 1 | 0 |
| Nausea | 1 | 0 |
| Shortness of breath | 1 | 0 |
| Dizziness | 1 | 0 |
| Coughing | 1 | 0 |
| **Ehrmann et al** | APP (564) | Control (557) |
| Skin breakdown | 8 | 10 |
| Vomiting | 15 | 18 |
| Central or arterial line dislodgement | 26 | 17 |
| Cardiac arrest at any time | 3 | 1 |
| **Fralick et al** | APP (126) | Control (122) |
| Aspiration pneumonia | 2 | 1 |
| Venous thromboembolism | 3 | 2 |
| Adverse events | 0 | 0 |
| **Harris et al** | APP (31) | Control (30) |
| Desaturation | 2 | 0 |
| Back pain | 3 | 0 |
| **Jayakumar et al** | APP (30) | Control (30) |
| Adverse events | 0 | 0 |
| **Nasrallah et al** | APP (45) | Control (45) |
| General discomfort & back pain | 10 | 0 |
| **Rosen et al** | APP (39) | Control (36) |
| Pressure sores | 9 | 2 |
| Vomiting | 0 | 1 |
| Central or arterial line dislodgement | 0 | 0 |
| Cardiac arrest within 30 days | 1 | 2 |
| **Liu et al** | APP (205) | Control (204) |
| Nausea | 2 | 3 |
| Unintentional removal of intravenous access | 0 | 0 |
| Pressure ulcer | 6 | 8 |
| Sacrococcygeal region | - | 8 |
| Face | 6 | - |
| Unexpected respiratory or cardiac arrest | 0 | 0 |

Supplement table 5 - Certainty of Evidence using the Grading of Recommendations, Assessment, Development, and Evaluation (GRADE) Approach.

| **Awake prone position compared to Usual care in adult patients with ARDS/AHRF** | | | | | | | | | | | |
| --- | --- | --- | --- | --- | --- | --- | --- | --- | --- | --- | --- |
| **Certainty assessment** | | | | | | | **Summary of findings** | | | | |
| **Participants (studies) Follow-up** | **Risk of bias^a^** | **Inconsistency** | **Indirectness** | **Imprecision** | **Publication bias** | **Overall certainty of evidence** | **Study event rates (%)** | | **Relative effect (95% CI)** | **Anticipated absolute effects** | |
|  |  |  |  |  |  |  | **With Usual care** | **With Awake prone position** |  | **Risk with Usual care** | **Risk difference with Awake prone position** |
| **Intubation** | | | | | | | | | | | |
| 3516 (20 RCTs) | not serious | not serious | not serious | not serious | none | ⨁⨁⨁⨁ High | 446/1715 (26.0%) | 364/1801 (20.2%) | **RR 0.80** (0.72 to 0.90) | 446/1715 (26.0%) | **52 fewer per 1,000** (from 73 fewer to 26 fewer) |
| **Mortality** | | | | | | | | | | | |
| 3588 (21 RCTs) | not serious | not serious | not serious | not serious | none | ⨁⨁⨁⨁ High | 314/1751 (17.9%) | 275/1837 (15.0%) | **RR 0.86** (0.74 to 0.99) | 314/1751 (17.9%) | **25 fewer per 1,000** (from 47 fewer to 2 fewer) |
| **Escalation of respiratory support** | | | | | | | | | | | |
| 2342 (13 RCTs) | not serious | serious^b^ | not serious | not serious | none | ⨁⨁⨁◯ Moderate^a^ | 335/1150 (29.1%) | 308/1192 (25.8%) | **RR 1.02** (0.81 to 1.30) | 335/1150 (29.1%) | **6 more per 1,000** (from 55 fewer to 87 more) |
| **Need for ICU admission** | | | | | | | | | | | |
| 575 (12 RCTs) | not serious | not serious | not serious | not serious | none | ⨁⨁⨁⨁ High | 66/272 (24.3%) | 65/303 (21.5%) | **RR 0.90** (0.65 to 1.24) | 66/272 (24.3%) | **24 fewer per 1,000** (from 85 fewer to 58 more) |

**CI:** confidence interval; **MD:** mean difference; **RR:** risk ratio

Explanations

a. Due to the nature of intervention, there was no blinding and was not considered as risk of bias

b. There was some heterogeneity for the outcome with I^2^ of 39%.
